# Cytotoxicity and Chemotaxonomic Significance of Saponins from Wild and Cultured *Asparagus* Shoots

**DOI:** 10.3390/molecules29143367

**Published:** 2024-07-18

**Authors:** Tarik Chileh-Chelh, Rosalía López-Ruiz, Ana M. García-Cervantes, Ignacio Rodríguez-García, Miguel A. Rincón-Cervera, Mohamed Ezzaitouni, José L. Guil-Guerrero

**Affiliations:** 1 Food Technology Division, University of Almería, 04120 Almería, Spain; chileh@hotmail.es (T.C.-C.); mrc883@ual.es (M.A.R.-C.); mohamedezzaitouni6@gmail.com (M.E.); 2 Department Chemistry-Physics, Analytical Chemistry of Contaminants, University of Almería, 04120 Almería, Spain; rosalialr@ual.es; 3 Department Chemistry-Physics CIAIMBITAL, University of Almería, 04120 Almería, Spain; agc874@inlumine.ual.es (A.M.G.-C.);; 4 Institute of Nutrition and Food Technology, University of Chile, Santiago 7830490, Chile

**Keywords:** *Asparagus*, saponins, LC-MS, HT-29 cells, MTT

## Abstract

The shoots of *Asparagus* L. are consumed worldwide, although most species belonging to this genus have a restricted range, and several taxa remain unstudied. In this work, a total of four taxa from different locations were scrutinized and compared with cultivated *A*. *officinalis*. All shoots were screened for saponins via LC-MS, and in vitro antiproliferative activities against the HT-29 colorectal cancer cell line were assessed via the MTT assay. The total saponins (TS) contained in the crude extracts ranged from 710.0 (*A. officinalis*) to 1258.6 mg/100 g dw (*A. acutifolius*). The richness of the compounds detected in this work stands out; a total of 47 saponins have been detected and quantified in the edible parts (shoots) of five taxa of *Asparagus*. The structure of all the saponins found present skeletons of the furostane and spirostane type. In turn, the structures with a furostane skeleton are divided into unsaturated and dioxygenated types, both in the 20–22 position. The sum of dioscin and derivatives varied largely among the studied taxa, reaching the following percentages of TS: 27.11 (*A. officinalis*), 18.96 (*A. aphyllus*), 5.37 (*A. acutifolius*), and 0.59 (*A. albus*); while in *A. horridus*, this compound remains undetected. Aspachiosde A, D, and M varied largely among samples, while a total of seven aspaspirostanosides were characterized in the analyzed species. The hierarchical cluster analysis of the saponin profiles clearly separated the various taxa and demonstrated that the taxonomic position is more important than the place from which the samples were acquired. Thus, saponin profiles have chemotaxonomic significance in *Asparagus* taxa. The MTT assay showed dose- and time-dependent inhibitory effects of all saponins extracts on HT-29 cancer cells, and the strongest cell growth inhibition was exercised by *A*. *albus* and *A*. *acutifolius* (GI_50_ of 125 and 175 µg/mL). This work constitutes a whole approach to evaluating the saponins from the shoots of different *Asparagus* taxa and provides arguments for using them as functional foods.

## 1. Introduction

Wild edible plants (WEPs) have been an essential component of human diets across cultures and continents for centuries. These plants offer a diverse array of essential nutrients, including vitamins, minerals, dietary fiber, micronutrients, and phytochemicals. In fact, they are often rich in antioxidants and therefore valuable contributors to human nutrition [1]. Their incorporation into diets can enhance nutritional diversity and address deficiencies in conventional diets, promoting better health outcomes.

The use of WEPs is deeply rooted in cultural traditions and practices. Indigenous and local communities have developed extensive knowledge about the identification, harvesting, and preparation of these plants, passing this wisdom down through generations. The food use of WEPs reflects cultural heritage, connects people to their natural surroundings, and strengthens cultural identity [2]. Moreover, WEPs serve as a valuable safety net during times of food scarcity and play a crucial role in enhancing food security. These plants are often resilient to environmental stressors and can thrive in diverse ecosystems, providing a reliable source of nutrition, especially in regions with limited access to conventional crops. Then, incorporating WEPs into agricultural systems can contribute to sustainable farming practices. Many of these plants require minimal input and are well-suited to organic and agroecological approaches. Their cultivation can enhance biodiversity, reduce the environmental impact of agriculture, and foster resilient food systems [3,4].

*Asparagus* is a genus in the Liliaceae family that includes over 250 species, from which *Asparagus officinalis* L. is the only cultivated species. However, there are several wild species that are traditionally collected for consumption and medicinal purposes in the Mediterranean Basin, such as *A. acutifolius* L. and *A. horridus* L. (syn. *A. stipularis* Forssk) [5]. *A. officinalis* is a widely cultivated vegetable with a long history of use as both food and medicine. The saponins content of the roots of *A. officinalis* has been extensively studied. In addition, the shoots of this plant have also been found to contain significant amounts of saponins, which are structurally similar to those found in the roots. Such compounds are integrated by a triterpenoid aglycone, such as protodioscin or dioscin, which is attached to one or more sugar residues. The sugar moieties are typically glucose, rhamnose, or xylose, and are linked to the aglycone through an ether bond. However, the exact composition of the saponins in *Asparagus* shoots can vary depending on the variety of the plant and environmental variables [6].

For instance, protodioscin was reported in the white shoots of *A. officinalis* by Lee at al. [7] at 1.4–5 mg/100 g fw, while Wang et al. [8] reported this saponin in the green shoots of the same species at 0.024–2.5 g/100 g fw. Concerning *A. acutifolius*, the saponins HTSAP-1, -3, -6, and ACSAP-1 were reported [9].

The saponins present in *Asparagus* shoots, including both *A. officinalis* and *A. acutifolius*, exhibit a wide range of biological activities, including anti-inflammatory, antioxidant, antitumor, antifungal, lipase inhibitory, antimicrobial, and immunomodulatory ones. However, further research is needed to fully understand the mechanisms of action of these compounds and to determine the optimal dosages and administration methods [9,10,11]. Some studies have also suggested that saponins may have a role in the promotion of healthy digestion and the regulation of blood lipid levels [12,13].

Wild *Asparagus* shoots are a rich source of saponins, which have been found to possess a range of biological activities and potential health benefits. Although the phytochemistry and phytotherapy of cultivated *A. officinalis* and wild *A. acutifolius* are relatively well known, there is a lack of knowledge on the saponins and biological activities of *A. stipularis*, *A. albus*, and *A. aphyllus* young shoots. All these species have recently been cited as sources of flavonoids (rutin, quercetin, nicotiflorin, asterin, and narcissine) and vitamin C, while their phenolic extracts exert selective antitumor activity against HT-29 colorectal cancer cells, especially those of *A. albus* [14]. Moreover, the variability of saponins composition of the various *Asparagus* taxa, depending on their ecogeographic location, constitutes research yet to be carried out. Therefore, the aim of this work is the determination of the saponin profiles of the edible shoots of 5 *Asparagus* species and their antitumor activities, to unravel their health benefits.

## 2. Results

### 2.1. Moisture Content

Considering all samples, the moisture content ranged from 81.4 in *A. acutifolius* AC1 to 91.1 g/100 g in *A. officinalis* O1. Focusing on the mean values of species, the amount was between 84.6 (*A. horridus*) and 91.0 g/100 g (*A. officinalis*).

### 2.2. Total Saponins and Saponin Profiles

Total saponins (Table 1) ranged in samples from 669.1 (*A. officinalis* O2) to 1529.3 mg/100 g dw (*A. acutifolius* AC2). As for species, values ranged from 710.0 (*A. officinalis*) to 1258.6 mg/100 g dw (*A. acutifolius*). Considering fresh weight, amounts ranged from 63.9 (*A. officinalis*) to 157.2 mg/100 g (*A. horridus*).

**Figure 1:**
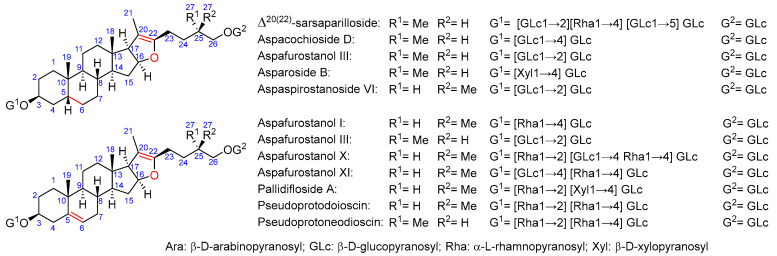
*Asparagus* saponins with furostane structure with an insaturation in C20–C22.

**Figure 2:**
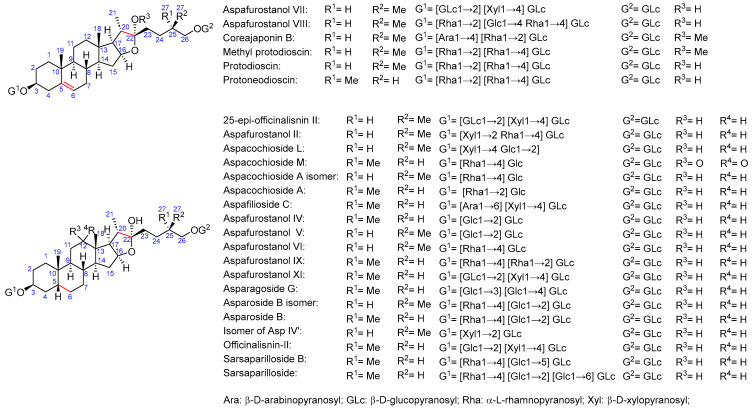
*Asparagus* saponins with furostane structure dioxygenated at C22.

**Figure 3:**
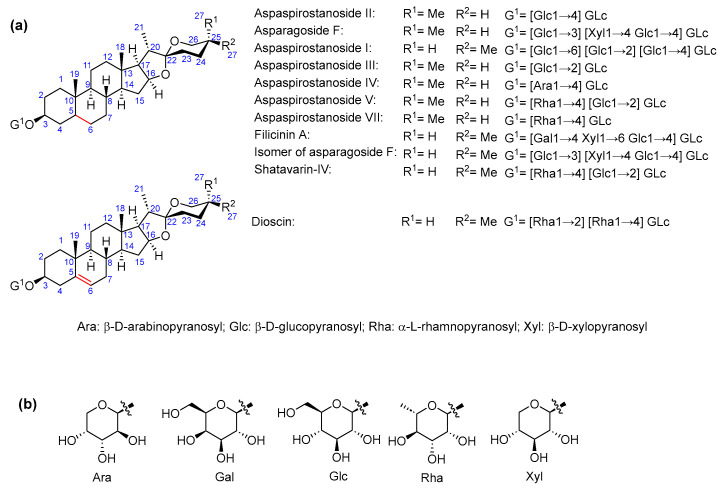
(**a**) *Asparagus* saponins with spirostane structure; (**b**) common sugars in all structures.

As for saponin profiles (Supplementary Table S1), a total of 47 different compounds were detected among the various samples. The sapogenin core of these compounds can be classified as furostane or spirostane. In turn, the structures with a furostane skeleton can be divided into unsaturated or dioxygenated types, both at the 20–22 position (Figure 1, Figure 2 and Figure 3). All the structures are detailed in Supplementary Table S2.

**Figure 4:**
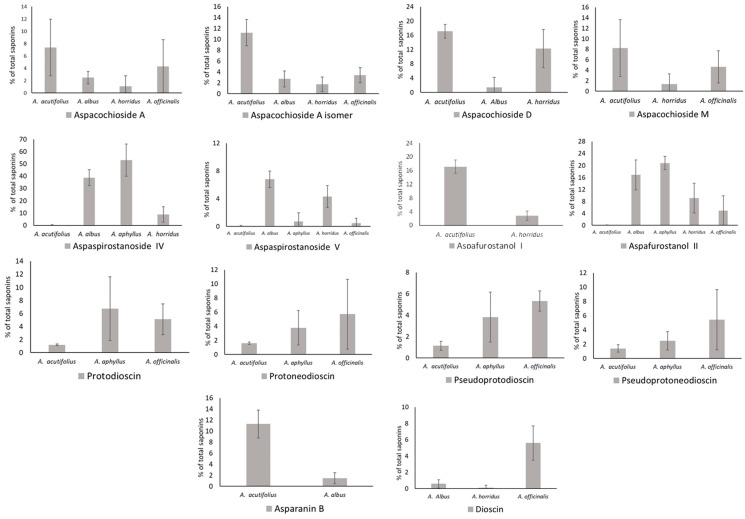
Occurrence of the main saponins detected in *Asparagus* shoots.

The occurrence of all these molecules is detailed in Supplementary Table S1, while the amounts of the main saponins are depicted in Figure 4. Aspachiosde A and Aspachioside A isomer were absent in *A. aphyllus*, and *A. acutifolius* showed the highest values, i.e., 7.37 and 11.24% of total saponins (TS). Aspacochioside D was present in *A. acutifolius* (17.12), *A. albus* (1.40), and *A. horridus* (12.33% TS), and Aspacochioside M occurs in *A. acutifolius* (8.13), *A. horridus* (1.32), and *A. officinalis* (6.79% TS). Dioscin was found in *A. albus*, *A. horridus*, and *A. officinalis*, reaching significant values only in the latter, i.e., 5.61% TS. Dioscin derivatives (protodioscin, protoneodioscin, pseudoprotodioscin, and pseudoprotoneodioscin) were detected in *A. acutifolius*, *A. aphyllus*, and *A. officinalis*. Protodioscin highlights in *A. aphyllus* (6.73) and *A. officinalis* (5.11% TS); protoneodioscin ranged from 1.66 (*A. acutifolius*) to 5.72% TS (*A. officinalis*); pseudoprotodioscin ranged between 1.14 (*A. acutifolius*) and 4.56% TS (*A. officinalis*); and pseudoprotoneodioscin ranged from 1.41 (*A. acutifolius*) to 5.44% TS (*A. officinalis*). Asparanin B was detected only in *A. acutifolius* and *A. albus* at 11.30 and 1.49% TS. A total of seven aspaspirostanosides were characterized in the analyzed species. From these, it reached noticeable amounts of aspaspirostanoside IV, which was absent in *A. officinalis* and ranged from 0.22 (*A. acutifolius*) to 53.03% TS (*A. aphyllus*), and aspaspirostanoside V, which occurs in all species, ranging from 0.05 (*A. acutifolius*) to 6.81% TS (*A. albus*). As for aspafurostanols, the I one was present in *A. acutifolius* (17.14) and *A. horridus* (2.83% TS), while the II one was found in all species, ranging from 0.04 in *A. acutifolius* to 20.84 in *A. aphyllus*. Some saponins were restricted to one single species; for instance, filicin A was detected only in *A. horridus* (11.80%), while aspafurostanols VIII-XI were restricted to *A. officinalis*, ranging from 5.31 (aspafurostanol VIII) to 10.43% TS (aspafurostanol XI).

### 2.3. Cluster Analysis

**Figure 5:**
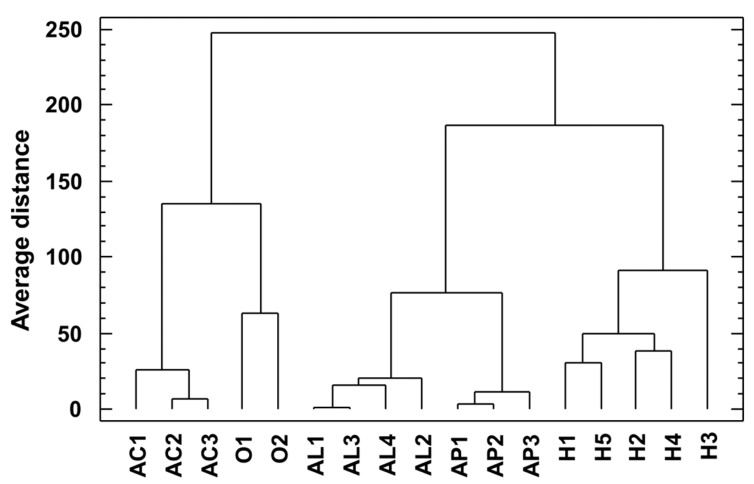
Dendrogram obtained from a cluster analysis of saponin profiles of sampled *Asparagus* species. Samples were clustered using Ward’s technique based on the city block distance measure.

Figure 5 shows the dendrogram obtained from a cluster analysis of all detected saponin profiles of sampled *Asparagus* species. Samples were clustered using Ward’s technique based on the city block distance measure.

### 2.4. Antiproliferative Activity

**Figure 6:**
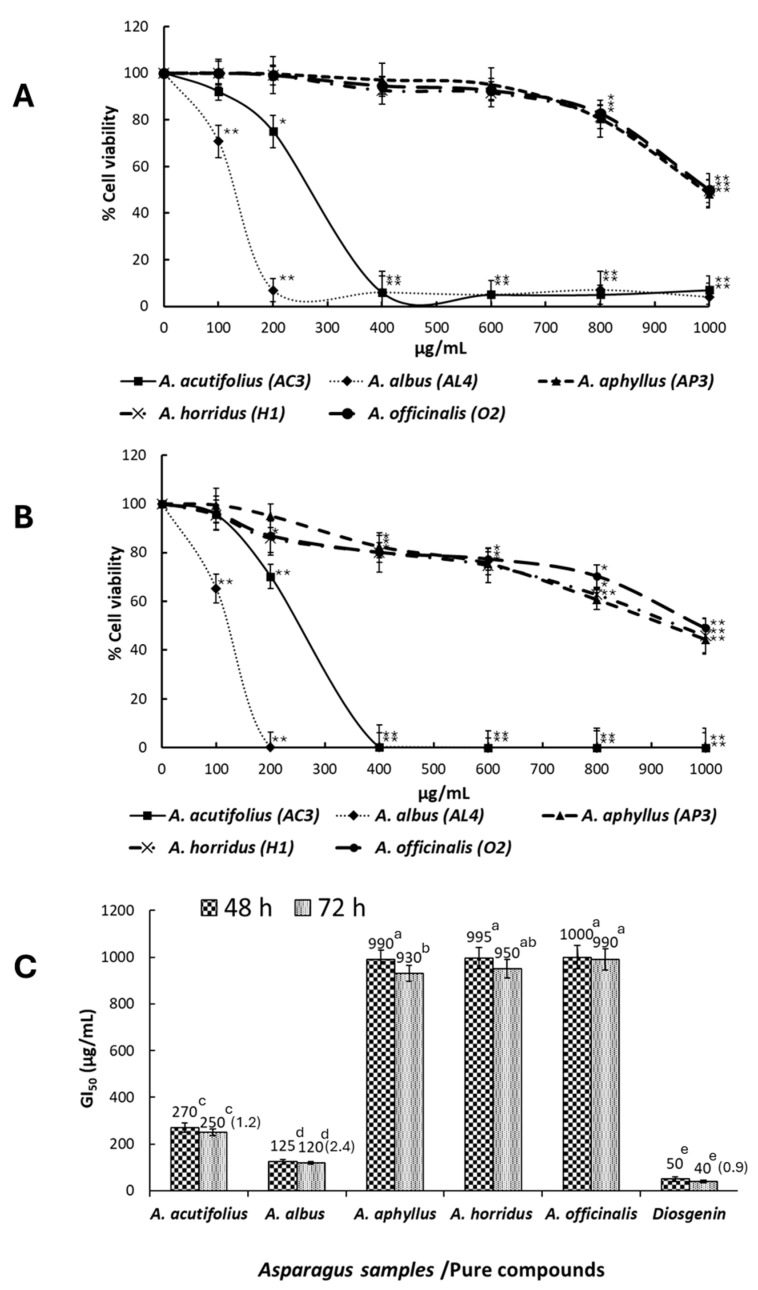
MTT assay. (**A**) Dose–response curves of HT-29 cell viability after treatment with different concentrations of saponin extracts of *Asparagus* samples for 48 h. (**B**) Dose–response curves of HT-29 cell viability after treatment with different concentrations of saponin extracts of *Asparagus* samples for 72 h. The statistical significance was evaluated at *p* < 0.05 (*) and *p* < 0.001 (**). (**C**) GI_50_ of HT-29 cells after cell treatment with saponin extracts of *Asparagus* samples and diosgenin for 48 and 72 h. The GI_50_ value is detailed over columns, and the Selectivity Index (SI) for 72-h exposed cells (HT-29 vs. CCD-18) to saponin extracts is shown in parentheses. Data represent the mean of three complete independent experiments ± SD (error bars). In a bar, means followed by different letters are significantly different at *p* < 0.05.

The MTT assay was accomplished to evaluate the inhibitory effects of extracts-containing *Asparagus* saponins on HT-29 human colorectal cancer cell viability. Extracts having the highest antioxidant activity (one of each species) were selected for this assay. Figure 6A,B show the activity of such extracts against HT-29 cancer cell viability after 48 and 72 h of treatment. Cell growth inhibition was exercised much better by extracts from *A. albus* and *A. acutifolius*, which, at 400 µg/mL and after 48 h of cell exposure to extracts, induced 6.9 and 6.3% of cell viability, and at 72 h of cell exposure, induced 0.3 and 0.1% of cancer cell viability in comparison with controls without extract addition. GI_50_ values—i.e., the doses of extracts that inhibited cell growth by 50%—for all samples are shown in Figure 6C. After a 72 h incubation period, GI_50_ for *A. acutifolius* (AC3), *A. albus* (AL4), *A. aphyllus* (AP3), *A. horridus* (H1), and *A. officinalis* (O2) were 250, 120, 930, 950, and 999 µg/mL, respectively. GI_50_ for diosgenin after 48 and 72 h of cell exposure to extracts were 50 and 40 µg/mL. The SI of HT-29 cancer cells versus CCD-18 normal cells was evaluated at 72 h of cells exposure only for extracts having GI_50_ ≤ 250 µg/mL, and it ranged from 1.2 (*A. acutifolius*) to 2.4 (*A. albus*), while for diosgenin, it was 0.9.

## 3. Discussion

### 3.1. Saponin Profiles of the Various Asparagus Species Analyzed in This Work

The total saponins-containing crude extract ranged from 710.0 (*A. officinalis*) to 1258.6 mg/100 g dw (*A. acutifolius*). These results are in line with those of Shao et al. [15], who obtained 1.72 g/100 g dw from *A. officinalis* shoots (gravimetrically determined). Vázquez-Castilla et al. [16] reported very low amounts in shoots: 10.9–27.3 mg/kg fw of Huétor *Asparagus*. Concerning *A. acutifolius*, Hamdi et al. [9] reported total saponins at 1419 mg/kg dw.

*Asparagus* saponins are steroidal glycosides. In *A. officinalis* and most of the green and white commercial hybrids derived from this specie, the main saponin is protodioscin (C_51_H_84_O_22_), which is a glycoside derivative of the furostanoid type diosgenin [13]. To date, more than 20 saponin aglycones have been identified in the genus *Asparagus*; however, only sarsasapogenin, asparanin A, protodioscin, yamogenin, and their derivatives have been studied [17]. The richness of compounds detected in this work stands out. A total of 47 saponins have been detected and quantified in the edible parts (shoots) of 5 taxa of wild *Asparagus* and farmed *A. officinalis*.

The sum of dioscin and derivatives, i.e., protodioscin, protoneodioscin, pseudoprotodoioscin, pseudoprotoneodioscin, and methyl protodioscin, varied largely among the studied taxa. It reached the following percentages of TS: 27.11 (*A. officinalis*), 18.96 (*A. aphyllus*), 5.37 (*A. acutifolius*), and 0.59 (*A. albus*); while in *A. horridus*, these compounds were undetected. Interestingly, diosgenin, a protodioscin moiety, was not found.

### 3.2. Multivariable Analyses for Assessing Chemotaxonomy

Figure 2 shows the dendrogram obtained from a cluster analysis of saponin profiles of sampled *Asparagus* species, where these are clearly separated. From these results, it is evident that the taxonomic position is more important than the place from which the samples were acquired. *Asparagus* is a complex genus in which a notable disagreement between molecular phylogeny and morphological taxonomy has been reported; for instance, several species belong to larger species complexes, both paraphyletic and polyphyletic ones. Thus, species delimitation should be based on both molecular and morphological data [18].

Four species analyzed belong to the Asparagus subgenus: *A. acutifolius*, *A. aphyllus*, *A. horridus*, and *A. officinalis*; while *A. albus* belongs to the Asparagopsis subgenus. In the obtained dendrogram, a close position was obtained for *A. horridus* and *A. aphyllus*, which were previously typified as genetically related [18]. However, *A. albus* was close to *A. acutifolius*, although a clear relationship between both species has not been reported yet. Probably, this fact is due to the absence of other members of the Asparagopsis subgenus in this analysis, and this fact is worthy of further research before confirming the utility of *Asparagus* saponins as a chemotaxonomical tool for subgenus *Asparagus* classification.

### 3.3. Antiproliferative Activity of the Saponins Extracts of Asparagus Shoots on HT-29 Cancer Cells

The saponins from *Asparagus* spp. have long been characterized as having antitumor activity. For instance, the crude saponins extract from the shoots of *A. officinalis* were cytostatic and cytocidal against the human leukemia HL-60 cells, and they inhibited the synthesis of DNA, RNA, and proteins [15].

Reports indicated that cytotoxic activity is characteristic of each *Asparagus* organ. Overall, the ethanolic extracts of rhizome and leaf are cytotoxic; however, low activity has been described for shoot extracts [9]. The rhizome extracts from several *Asparagus* species were tested against the HepG2 (liver cancer) cell line. Three *Asparagus* species, namely, *A. acutifolius* [9], *A. adscendent* [19], and *A. filicinius* [20], exercised noticeable cytotoxic activity, and this has been related to the occurrence of saponins and their sapogenins. However, the rhizome extract of *A. albus* showed low activity [21].

Some pure saponins isolated from *Asparagus* spp. have been tested against cancer cells. For instance, asparanin A, a steroidal saponin, exhibited anticancer activity on endometrial cancer. This saponin inhibited cell proliferation and caused cell morphology alteration and cell cycle arrest in the G0/G1 phase, apoptosis through the mitochondrial pathway, generation of ROS, and activation of caspases, alongside other mechanisms. It inhibited tumor cell proliferation and growth in vivo and induced apoptosis [22]. Asparanin A also induces cell cycle arrest and triggers apoptosis via a p53-independent manner in HepG2 cells [23].

Considering the activity against colorectal cancer cells, the saponins from *Asparagus* have been typified as inhibitors through cytotoxicity and apoptosis [24]. For instance, the saponins from edible spears of wild asparagus (triguero Huétor-Tájar, HT, landrace) inhibit AKT, p70S6K, and ERK signaling and induce apoptosis through G0/G1 cell cycle arrest in human colon cancer HCT-116 cells [25]. Both the rhizome and leaf from *A. acutifolius* showed high activity against this cell line, while the leaf extracts from *A. albus* and *A. acutifolius* species had similar IC_50_ values for HCT-116 cells [21]. Interestingly, when checking the rhizome extract of *A. officinalis* against HCT-116 cells, the IC_50_ value was better than that of the saponins extracted from the corresponding by-products [26], and this result was related to a different saponin compositions or to the synergistic effects among the various phytochemicals present in *A. acutifolius* extracts.

Zhao et al. [27] reported the activity of the saponins-containing crude extract against the colon cancer cell lines SW620 and HCT-116 through the induction of cytotoxicity. Jaramillo-Carmona et al. [28] found that protodioscin induced cytotoxicity in HCT-116, HT-29, and Caco-2 colon cancer cells. Dioscin exercises antitumor activities against several types of tumors, such as lung cancer, gastric cancer, colon cancer, glioblastoma, cervix carcinoma, ovarian cancer, breast cancer, prostate cancer, and leukemia. Its antitumor activity is exercised through intrinsic mitochondrial apoptosis, involving activation of caspase-9 and caspase-3, and induces a reduction in antiapoptotic proteins such as Bcl-2, Bcl-xl, cIAP-1, and Mcl-1 [29].

In this work, after 48 and 72 h of treatment, the MTT assay revealed concentration- and time-dependent inhibitory effects on HT-29 cells for all assayed extracts (Figure 6A,B). The antitumor activity was especially intense for the extracts obtained from the stems of *A. albus* and *A. acutifolius*. In the case of *A. albus*, it contains saponins that, in descending order, are aspaspirostanoside IV, aspafurostanol II, and aspaspirostanoside V. However, considering that these same saponins are found in *A. aphyllus*, which develops low activity against HT-29 cells, it is difficult to attribute the observed activity to such saponins. This is not the case of *A. acutifolius*, which contains characteristic saponins, such as aspafurostanol I, asparanin B (shatavarin-IV), and aspachoioside M, which could have exerted the noted action. Interestingly, these two highly active species are the only ones that contain shatavarin IV, especially *A. acutifolius*. This saponin was previously isolated from *A. racemosus* roots [30]. The cytotoxicity (in vitro) of shatavarin IV extracts (approximately 5% of shavaratins) and other shatavarins rich fraction was assayed by the MTT test against HT-29 cells, showing significant anticancer activity in both in vitro and in vivo experimental models [30]. Therefore, considering the content of shavaratin IV (11.30 in *A. acutifolius* and 1.49% in *A. albus*), it is likely that the noted activity was due to this saponin type, at least partially.

Although *A. officinalis* shows high percentages of dioscin and its derivatives, the activity of its crude extract against HT-29 cells was very week; a possible explanation is that this cell line is weakly sensitive to these saponin types.

The National Cancer Institute (NCI) consider compounds/extracts/fractions as cytotoxic when their GI_50_ values are within the 20–30 µg/mL range [31]. Based on the MTT results, the saponin extracts checked lack cytotoxicity, while diosgenin was recognized as cytotoxic to the tested cell line. It should be noted that the extracts tested are not completely made up of saponins; therefore, it is quite possible that the isolation of pure saponin fractions from these extracts will yield cytotoxic compounds. The GI_50_ value of diosgenin for the HT-29 cell line obtained in the current work via the MTT assay is consistent with previous studies for such a compound on the HeLa cancer cell line (e.g., [32]). On the other hand, according to the threshold proposed by Suffness and Pezzuto [33], crude extracts showing a GI_50_ ≤ 100 µg/mL can be selected for further studies, whereas the most promising ones are those with a GI_50_ < 30 µg/mL. Thus, the saponin extract from *A. albus* shoot, whose GI_50_ is close to this figure (120 µg/mL), merit further research for its fractionation until pure active compounds can be isolated. Then, the mechanisms of action of such compounds against different cancer cell lines would be checked according to different methodologies.

It should be noted that the antiproliferative effects of the extracts may not be due to specific compounds. It is likely that interactions among the various saponins and with other components present in the extracts could contribute to the overall reported effects. In this regard, the anticancer effects of a deproteinized *Asparagus* polysaccharide on hepatocellular carcinoma cells have been reported. This *Asparagus* polysaccharide exerts both an effective inhibitor effect on cell growth in vitro and in vivo and also exerts potent selective cytotoxicity against human hepatocellular carcinoma Hep3B and HepG2 cells. Such a polysaccharide develops activity through an apoptosis-associated pathway by modulating the expression of Bax, Bcl-2, and caspase-3 and has been proposed as a potential therapeutic agent (or chemosensitizer) for liver cancer therapy [34].

## 4. Materials and Methods

### 4.1. Samples

Shoots were collected in the locations listed in Table 2 or purchased in local markets. Upon arrival at the laboratory, the shoots were labeled, weighed, measured, and placed in a glass desiccator until analysis. Just prior to analysis, shoots were ground to powder with a mortar. Approximately 2 g of each sample was used for moisture analysis, which was carried out in a forced air oven at 105 °C for 8 h. All results are reported on a dry weight (dw) basis.

### 4.2. Extraction of Saponins

Sample preparation was carried out in triplicate to obtain the various *Asparagus* extracts. A weight of 0.5 g of wet *Asparagus* sample was suspended in 20 mL of dichloromethane. The mixture was sonicated in an ultrasonic bath set at 50 °C for 5 min and then filtered. The solid residue was subjected to the same extraction procedure with dichloromethane once more. Next, the residue was solubilized with 20 mL of methanol and extracted with an ultrasonic bath set at 50 °C for 5 min and filtrated. The residue was subjected to the same extraction procedure with methanol one last time. Combined methanolic filtrates were evaporated under reduced pressure with a rotary evaporator until dryness was attained. Solid methanolic extracts were redissolved in methanol (10 mg/mL) and centrifugated at 10.000× *g* for 5 min, and the supernatants were used for analysis [35].

### 4.3. Total Saponin Content

TS of the *Asparagus* extracts was determined using a spectrophotometric method as described by Ncube et al. [36] with minor modifications. Briefly, the dried methanolic extracts previously obtained were prepared at 10 mg/mL in methanol. Aliquots of 125 μL were transferred to vials, followed by 125 μL of a freshly prepared solution of vanillin in ethanol (0.8%, *w*/*v*) and 1.25 mL of sulfuric acid in water (72%, *v*/*v*). A control sample using methanol was also prepared. Samples were vortexed and heated at 60 °C for 10 min. Vials were cooled in ice for 5 min, and absorbance was measured at 520 nm using a UV-VIS spectrophotometer against the control sample containing methanol. TS was obtained from a standard curve of diosgenin using solutions ranging from 100 to 1700 μg/mL, which were prepared under the same conditions as previously stated for samples. Diosgenin was used as a representative standard of steroid saponins. Results were expressed as mg of total saponins per 100 g of dry sample. Determinations were undertaken in triplicate.

### 4.4. Characterization of Saponins by LC-MS

This methodology is fully detailed in Supplementary File S1, while data related to the determination of saponins by LC-MS are provided in Supplementary Table S3. The chromatographic separations were performed on a Vanquish Flex Quaternary LC equipped with a reverse-phase C18 column (Hypersil Gold, 100 mm × 2.1 mm, 1.9 μm) at a flow rate of 0.3 mL/min. The compounds were separated with gradient elution using acidified water (H_2_O containing 0.1% formic acid) (A) and acetonitrile (B) as eluents at room temperature (30 °C). The LC system is coupled to a single mass spectrometer Orbitrap Thermo Fisher Scientific (Exactive^TM^, Thermo Fisher Scientific, Bremen, Germany) using an electrospray interface (ESI) (HESI-II, Thermo Fisher Scientific, San Jose, CA, USA) in positive and negative ion mode. Mass range in the full scan experiments was set at *m/z* 90–1000. LC chromatograms were acquired using the external calibration mode, and they were processed using Xcalibur^TM^, version 3.0, with Qualbrowser and Trace Finder 4.0 (Thermo Fisher Scientific, Les Ulis, France). Unknown analysis was carried out with Compound Discoverer^TM^, version 2.1.

### 4.5. Antitumor Assays

This methodology is fully detailed in Supplementary File S1. The antiproliferative activity of the saponin extracts from *Asparagus* shoots were assayed on the HT-29 human colon cancer cell line and the CCD-18 colonic human myofibroblasts cells line as described by Lyashenko et al. [37]. Cell cultures were supplied by the Technical Instrumentation Service of the University of Granada (Granada, Spain).

### 4.6. Statistical Analysis

Three aliquots for each sample were analyzed in triplicate for each location to obtain the results of saponins, and all data in the tables are reported as the mean value ± SD. The significance of the differences among the mean values was assessed via one-way ANOVA coupled with Fisher’s LSD test at *p* < 0.05. Pearson product–moment correlation (r) and statistical significance (*p*) were obtained for each pair of variables (the saponins). *p* < 0.05 was regarded as significant. Cluster analysis was performed using agglomerative hierarchical clustering (AHC) (Ward’s technique) based on the city block distance measure. All statistical analyses were carried out using Statgraphics^©^ centurion XVI (StatPoint Technologies, Warrenton, VA, USA).

## 5. Conclusions

In this work, a total of 47 saponins were detected and quantified in the edible parts (shoots) of four taxa of wild *Asparagus* and cultured *A. officinalis*. The structure of all the saponins found contains skeletons of the furostane and spirostane type. The sum of dioscin and derivatives greatly varied among the studied taxa, and these, together with aspaspirostanosides (seven types detected) and aspachiosdes A, D, and M, constitute the larger fractions of the *Asparagus* saponins detected in this work. The hierarchical cluster analysis of the saponin profiles clearly separated the various taxa and demonstrated that the taxonomic position is more important than the place from which the samples were acquired. Thus, the saponin profiles have chemotaxonomic significance in *Asparagus* taxa. The MTT assay showed dose- and time-dependent inhibitory effects of all saponin extracts on HT-29 cancer cells, and *A. albus* and *A. acutifolius* exercised the strongest cell growth inhibition after a 72-h period of cells exposure to extracts. Given the richness in saponins and antitumor activities, most *Asparagus* taxa analyzed here have the potential to be used as functional foods. Further research involving the purification of the various saponins fractions from several *Asparagus* extracts and one-to-one antitumor tests against several cancer cell lines could evidence their in vitro antiproliferative activity more clearly.

## Figures and Tables

**Table 1:** Moisture and saponins content of *Asparagus* samples ^1^.

Species/Samples	Moisture (g/100 g)	Saponins (mg/100 g Dry Weight)
*A. acutifolius*		
AC1	81.4 ± 0.0 ^h^	1094.9 ± 21.2 ^def^
AC2	84.6 ± 0.3 ^fg^	1529.3 ± 107.8 ^a^
AC3	85.2 ± 0.3 ^ef^	1151.5 ± 7.9 ^de^
Mean ± SD	84.5 ± 2.3 ^B^	1258.6 ± 236.2 ^A^
*A. albus*		
AL1	88.0 ± 0.2 ^bc^	1405.2 ± 176.3 ^ab^
AL2	88.1 ± 0.2 ^bc^	996.9 ± 39.1 ^efg^
AL3	86.4 ± 1.2 ^de^	1387.4 ± 107.9 ^abc^
AL4	89.0 ± 0.5 ^b^	930.5 ± 98.5 ^fgh^
Mean ± SD	87.9 ± 1.1 ^AB^	1180.0 ± 251.3 ^AB^
*A. aphyllus*		
AP1	83.7 ± 0.9 ^g^	907.8 ± 27.3 ^fgh^
AP2	85.3 ± 0.5 ^ef^	860.8 ± 115.0 ^gh^
AP3	86.5 ± 1.2 ^de^	909.3 ± 7.7 ^fgh^
Mean ± SD	85.2 ± 1.4 ^B^	892.6 ± 27.6 ^AB^
*A. horridus*		
H1	80.7 ± 1.4 ^h^	1211.5 ± 60.3 ^cd^
H2	84.0 ± 0.7 ^fg^	1422.9 ± 70.8 ^ab^
H3	83.3 ± 1.1 ^g^	838.3 ± 174.1 ^ghi^
H4	87.5 ± 0.1 ^cd^	838.1 ± 55.5 ^ghi^
H5	87.3 ± 0.5 ^cd^	794.9 ± 31.2 ^hi^
Mean ± SD	84.6 ± 2.9 ^B^	1021.1 ± 281.0 ^AB^
*A. officinalis*		
O1	91.1 ± 0.2 ^a^	750.9 ± 112.0 ^hi^
O2	90.9 ± 0.3 ^a^	669.1 ± 101.0 ^i^
Mean ± SD	91.0 ± 0.1 ^A^	710.0 ± 57.8 ^B^

^1^ Data represent means ± SD of samples analyzed in triplicate. Differences in saponin amounts were tested according to one-way ANOVA followed by Duncan’s test. Within a column, means followed by different lowercase letters are significantly different at *p* < 0.05, and means followed by capital letters represent the ANOVA test effected for mean values of species (*p*< 0.05).

**Table 2:** Data on samples collection of the shoots of *Asparagus* species.

Species/Location	Code	Geographical Coordinates	Date
*A. acutifolius* (Raviscanina)			
Punta Entinas, El Ejido, Almería	AC1	36.690831, −2.7732501	04 April 2023
Algámitas, Sevilla	AC2	37.018445, −5.161181	07 March 2023
Fonelas, Granada	AC3	37.409918, −3.200831	10 May 2023
*A. albus* (White asparagus)			
Darrícal, Almería	AL1	36.917674, −3.028230	17 March 2023
Sierra Cabrera, Almería	AL2	37.134984, −1.868005	05 January 2023
Barranco de las Lastras, Adra, Almería	AL3	36.790886, −3.100039	03 March 2023
El Toyo, Almería	AL4	36.847975, −2.332920	03 February 2023
*A. aphyllus* (Prickly asparagus)			
Zahara de la Sierra, Cádiz	AP1	36.841757, −5.395525	20 March 2023
Mijas, Málaga	AP2	36.591142, −4.606242	23 March 2023
Torremolinos, Málaga	AP3	36.605964, −4.526782	20 March 2023
*A. horridus* (Esparraguera)			
Cabo de Gata, Almería	H1	36.723495, −2.183220	19 March 2023
Vícar, Almería	H2	36.813587, −2.60462	11 March 2023
Enix, Almería	H3	36.875594, −2.609560	12 March 2023
Las Amoladeras Almería	H4	36.817729, −2.253485	07 March 2023
Rodalquilar, Níjar	H5	36.849231, −2.043093	14 February 2023
*A. officinalis* (Garden asparagus)			
Láchar, Granada	O1	Purchased	01 April 2023
Loja, Granada	O2	Purchased	05 October 2023

## Data Availability

All data concerning this research are available in the figures and tables of the article.

## Supplementary Materials

The following supporting information can be downloaded at https://www.mdpi.com/article/10.3390/molecules29143367/s1: Supplementary File S1. Material and Methods and Supplementary Tables: Supplementary Table S1. Saponin profiles of *Asparagus* shoots (individual saponin% of total saponin area reported by the LC-MS system); Supplementary Table S2. Saponins structures detected in the *Asparagus* shoots analized; and Supplementary Table S3. Data on saponins determination by LC-MS. References [38,39] are cited in the Supplementary Materials.
