# Supplementary material for: Cytotoxicity and Chemotaxonomic Significance of Saponins from Wild and Cultured Asparagus Shoots
Source: Molecules. 2024 Jul 18;29(14):3367. doi: 10.3390/molecules29143367 (PMC11279782; doi:10.3390/molecules29143367)
Supplement: Supplementary file 1 [file molecules-29-03367-s001.zip › S1-Material and Methods.pdf]

molecules

MDPI

Supplementary Materials

Cytotoxicity and Chemotaxonomic Significance of Saponins from Wild and Cultured Asparagus Shoots

Tarik Chileh-Chelh [1], Rosalía López-Ruiz [2], Ana M. García-Cervantes [3], Ignacio Rodríguez-García [3], Miguel A. Rincón-Cervera [1,4], Mohamed Ezzaitouni [1] and José L. Guil-Guerrero [1,*]

[1] Food Technology Division, University of Almería, 04120 Almería, Spain
[2] Dept. Chemistry-Physics, Analytical Chemistry of Contaminants, University of Almería, 04120 Almería, Spain
[3] Dept. Chemistry-Physics CIAIMBITAL, University of Almería, 04120 Almería, Spain
[4] Institute of Nutrition and Food Technology, University of Chile, Santiago 7830490, Chile
* Correspondence: jlguil@ual.es; Tel.: +34-640-225-578

Supplementary File 1. Material and Methods

1. Characterization of Saponins by LC-MS

The chromatographic separations were performed on a Vanquish Flex Quaternary LC equipped with a reverse-phase C18 column (Hypersil Gold, 100 mm × 2.1 mm, 1.9 µm) at a flow rate of 0.3 mL/min. The compounds were separated with gradient elution using acidified water (H_2O containing 0.1% formic acid) (A) and acetonitrile (B) as eluents at room temperature (30 ºC). The step gradient was as follows: 0-6 min 76% A; then, it was linearly decreased to 73% in 2 min, to 72% in 4 min, to 68% in 2 min, to 58% in 6 min, to 20% in 3 min and remained constant during 2 min. Later, it increased to 76% in 5 min and remained constant for 5 min. The total running time was 35 min. The injection volume was 10 µL.

The LC system is coupled to a single mass spectrometer Orbitrap Thermo Fisher Scientific (ExactiveTM, Thermo Fisher Scientific, Bremen, Germany) using an electrospray interface (ESI) (HESI-II, Thermo Fisher Scientific, San Jose, CA, USA) in positive and negative ion mode. ESI parameters were as follows: spray voltage, 4 kV; sheath gas (N_2 > 95%), 35 (adimensional); auxiliary gas (N_2 > 95%), 10 (adimensional); skimmer voltage, 18 V; capillary voltage, 35 V; tube lens voltage, 95 V; heater temperature, 305 °C; capillary temperature, 300 °C. Operating in Full Scan mode (mass resolving power of 70000 FWHM at m/z 200) and in data independent acquisition (DIA) mode (mass resolving power of 35000 FWHM at m/z 200) with HCD fragmentation with a collision energy (CE) of 30 eV and an isolation window of m/z 50. Mass range in the full scan experiments was set at m/z 90–1000. LC chromatograms were acquired using the external calibration mode and they were processed using XcaliburTM version 3.0, with Qualbrowser and Trace Finder 4.0 (Thermo Fisher Scientific, Les Ulis, France). Unknown analysis was carried out with Compound DiscovererTM version 2.1.

The LC coupled to Orbitrap MS is a powerful analytical technique used for identifying and quantifying saponins. Sensitivity and specificity are key performance parameters for this technique:

Sensitivity: Sensitivity refers to the ability of the LC-Orbitrap MS system to detect and quantify analytes at low concentrations. In LC-Orbitrap MS, sensitivity is often excellent due to the high-resolution capabilities of the Orbitrap mass analyzer and the sensitivity of modern LC systems. The instrument can detect analytes at very low concentrations, often in the low parts-per-billion (ppb) or even parts-per-trillion (ppt) range.

Specificity: Specificity refers to the ability of the LC-Orbitrap MS system to differentiate between analytes of interest and other compounds present in the sample matrix. The high resolution and mass accuracy of the Orbitrap mass analyzer contribute to excellent specificity by enabling precise determination of the mass-to-charge ratios (*m/z*) of analytes (up to 5 decimal points). Additionally, LC separation prior to MS analysis helps to resolve complex mixtures, further enhancing specificity.

2. Cell Assays

The anticancer activity was determined for saponin extracts from *Asparagus* shoots. The HT-29 colon cancer cells line and the CCD-18 colonic human myofibroblasts cells line were used to check antiproliferative activities. Cultures were supplied by the Technical Instrumentation Service of the University of Granada (Granada, Spain). First, they were checked for the absence of *Mycoplasma* and bacteria. Then, cells were grown at 37 °C and 5% CO_2 humidified atmosphere in medium RPMI-1640 supplemented with 5% fetal bovine serum, 2 mM L-Glutamine, 1 mM sodium pyruvate, 0.125 mg/mL amphotericin, and 100 mg/mL penicillin-streptomycin.

All cultures were plated in 25 cm^2 plastic tissue culture flasks (Sarstedt, Newton, NC, USA). Cell culture and cell assay, that is, the MTT test, were accomplished as previously described [1].

In the MTT assay, cells were divided into 96-well microtiter plates, adjusted to $1{\times}10^4$ cells/well, and cultivated in a medium at 37 °C and 5% CO_2 prior to adding the different

extracts dissolved in the medium. The saponin-containing extracts were supplied to cells dissolved in a mixture of DMSO and then in the culture medium at designed concentrations (0-1000 μg/mL). After 48 and 72 h of cell exposure, 5 mg/mL of an MTT solution was added to the culture medium to determine the viability of cells. The absorbance was recorded at 570 nm on an enzyme-linked immunosorbent assay (ELISA) plate reader (Thermo Electron Corporation, Sant Cugat del Valles, Barcelona, Spain). The formazan crystals produced were solubilized using 100 μL of DMSO. Cells without saponin extracts were considered negative controls, which were used for all concentrations and tested extracts. Cell survival in exposed cultures relative to unexposed cultures was calculated, and the number of viable cells was calculated using the following equation:

$$\text{Percentage of viable cells (\%)} = (\text{Absorbance of treated cells/Absorbance of untreated cells}) \times 100\% \quad (1)$$

The concentrations causing 50% cell growth inhibition (GI_{50}) were calculated from the growth curves. Diosgenin (99%, 700085P) from Merck (Madrid, Spain) was used as a positive control, while DMSO and methanol were used as the negative (vehicle) controls. Saponins extracts and controls were evaluated in three independent assays. Values presented are mean ± standard error of the mean. The SI of each compound was calculated as GI_{50} of the extract against the CCD-18 normal cell line/GI_{50} of the same extract against the HT-29 cancer cell line [2].

References

1. Ramos-Bueno, R.P.; Romero-González, R.; González-Fernández, M.J.; Guil-Guerrero, J.L. Phytochemical composition and*in vitro*anti-tumour activities of selected tomato varieties. *J. Sci. Food Agric.* **2017**, *97*, 488–496, https://doi.org/10.1002/jsfa.7750.
2. Vichitsakul, K.; Laowichuwakonnukul, K.; Soontornworajit, B.; Poomipark, N.; Itharat, A.; Rotkrua, P. Anti-proliferation and induction of mitochondria-mediated apoptosis by Garcinia hanburyi resin in colorectal cancer cells. *Heliyon* **2023**, *9*, e16411. https://doi.org/10.1016/j.heliyon.2023.e16411
