# Supplementary material for: Cytotoxicity and Chemotaxonomic Significance of Saponins from Wild and Cultured Asparagus Shoots
Source: Molecules. 2024 Jul 18;29(14):3367. doi: 10.3390/molecules29143367 (PMC11279782; doi:10.3390/molecules29143367)
Supplement: Supplementary file 1 [file molecules-29-03367-s001.zip › Supplementary Tables.pdf]

Supplementary Tables of the article:

Cytotoxicity and Chemotaxonomic Significance of Saponins from Wild and Cultured *Asparagus* Shoots

Supplementary Table S1. Saponin profiles of wild *Asparagus* shoots (individual saponin% of total saponin area reported by the LC-MS system) [a]

		A. Acutifolius				*A. albus*					*A. aphyllus*				*A. horridus*						*A. officinalis*		
**CAS number**	**Common name**	**AC1**	**AC2**	**AC3**	**Mean**	**AL1**	**AL2**	**AL3**	**AL4**	**Mean**	**AP1**	**AP2**	**AP3**	**Mean**	**H1**	**H2**	**H3**	**H4**	**H5**	**Mean**	**O1**	**O2**	**Mean**
1494664-30-0	25-epi-officinalisnin II	n.d.	n.d.	0.48±0.56^{b}	0.16±0.27^{B}	3.26±1.00^{a}	1.25±0.95^{ab}	3.30±1.09^{a}	3.42±1.07^{a}	2.81±1.04^{A}	n.d.	n.d.	n.d.	n.d.	2.64±0.80^{a}	1.20±1.76^{ab}	3.77±1.98^{a}	0.89±1.45^{b}	0.98±1.66^{b}	1.9±1.26^{A}	1.45±1.38^{ab}	4.13±3.09^{a}	2.79±1.90^{A}
89590-92-1	Asp VI	n.d.	n.d.	n.d.	n.d.	n.d.	n.d.	n.d.	n.d.	n.d.	3.07±1.09^{a}	4.51±1.05^{a}	3.36±1.65^{a}	3.65±0.76^{A}	n.d.	n.d.	0.50±1.78^{c}	n.d.	n.d.	0.10±1.04^{C}	1.56±1.99^{b}	1.15±1.09^{bc}	1.36±0.29^{B}
2417238-29-8	Aspacochinoside L	n.d.	n.d.	2.09±0.76^{c,d}	0.70±1.61^{C}	4.39±1.90^{bc}	6.06±1.75^{a}	5.64±2.04^{ab}	3.71±1.96^{bc}	4.96±1.13^{A}	n.d.	n.d.	n.d.	n.d.	2.64±1.76^{c}	3.93±1.78^{bc}	8.77±3.53^{a}	2.34±1.08^{cd}	0.98±1.80^{d}	3.73±3.00^{AB}	0.86±1.56^{d}	2.45±1.77^{cd}	1.66±1.44^{B}
557769-32-1	Aspacochioside A	9.02±2.09^{a}	2.18±1.41^{cd}	10.9±2.09^{a}	7.37±4.59^{A}	1.91±0.43^{cd}	3.95±1.17^{b}	1.91±1.68^{cd}	2.14±1.45^{cd}	2.48±0.99^{C}	n.d.	n.d.	n.d.	n.d.	n.d.	1.81±1.40^{cd}	n.d.	3.76±2.90^{bc}	n.d.	1.11±1.67^{C}	7.39±2.08^{a}	1.2±1.77^{d}	4.29±4.37^{B}
927890-95-7	Aspacochioside A isomer	9.02±1.99^{a}	13.8±1.45^{a}	10.9±2.99^{a}	11.24±2.41^{A}	1.91±1.90^{bc}	4.95±2.01^{b}	1.91±1.53^{bc}	2.14±0.77^{b}	2.73±1.48^{BC}	n.d.	n.d.	n.d.	n.d.	1.55±1.89^{c}	1.81±2.09^{c}	n.d.	3.76±1.06^{bc}	1.58±3.02^{c}	1.74±1.34^{C}	4.39±1.55^{b}	2.45±1.39^{bc}	3.42±1.37^{B}
1351930-52-3	Aspacochioside D	14.95±3.09^{b}	17.6±3.98^{ab}	18.8±2.99^{ab}	17.12±1.97^{A}	n.d.	5.60±1.80^{d}	n.d.	n.d.	1.40±2.80^{B}	n.d.	n.d.	n.d.	n.d.	10.61±2.67^{b}	13.39±4.08^{b}	6.90±2.04^{cd}	20.98±3.07^{a}	9.77±3.06^{bc}	12.33±5.36^{A}	n.d.	n.d.	n.d.
2417238-30-1	Aspacochioside M	14.39±2.08^{a}	5.25±0.99^{b}	4.76±1.87^{b}	8.13±5.43^{A}	n.d.	n.d.	n.d.	n.d.	n.d.	n.d.	n.d.	n.d.	n.d.	n.d.	4.41±2.76^{bc}	n.d.	2.17±1.90^{c}	n.d.	1.32±1.97^{C}	2.45±1.77^{c}	6.79±3.99^{b}	4.62±3.07^{BC}
131123-74-5	Aspafilioside C	n.d.	n.d.	n.d.	n.d.	n.d.	n.d.	n.d.	n.d.	n.d.	n.d.	n.d.	n.d.	n.d.	1.25±1.60	1.61±1.16	0.55±1.73	3.96±2.40	1.58±2.09	1.79±1.29	n.d.	n.d.	n.d.
117457-34-8	Aspafurostanol I	14.97±2.87^{a}	17.69±2.22^{a}	18.77±3.09^{a}	17.14±1.96^{A}	n.d.	n.d.	n.d.	n.d.	n.d.	n.d.	n.d.	n.d.	n.d.	3.26±3.05^{bc}	3.90±1.84^{b}	1.74±0.88^{c}	4.23±1.88^{b}	1.04±1.77^{c}	2.83±1.38^{B}	n.d.	n.d.	n.d.
270926-87-9	Aspafurostanol II	n.d.	0.12±0.15^{e}	n.d.	0.04±0.07^{E}	17.13±3.21^{b}	17.36±2.09^{b}	22.63±2.08^{a}	10.32±2.05^{c}	16.86±5.05^{B}	18.34±3.23^{ab}	22.04±3.65^{a}	22.15±3.09^{a}	20.84±2.17^{A}	7.30±2.09^{c}	9.54±3.08^{c}	3.15±1.77^{d}	16.75±3.67^{b}	8.64±2.88^{c}	9.08±4.94^{C}	8.39±2.75^{c}	1.34±1.44^{d}	4.86±4.98^{D}
185432-00-2	Aspafurostanol III	n.d.	n.d.	n.d.	n.d.	n.d.	n.d.	n.d.	n.d.	n.d.	n.d.	n.d.	n.d.	n.d.	n.d.	1.40±1.87^{a}	n.d.	n.d.	n.d.	0.28±0.63^{A}	n.d.	n.d.	n.d.
1193356-76-1	Aspafurostanol IV:	n.d.	n.d.	n.d.	n.d.	n.d.	n.d.	n.d.	n.d.	n.d.	n.d.	n.d.	n.d.	n.d.	n.d.	n.d.	n.d.	n.d.	n.d.	n.d.	n.d.	n.d.	n.d.
244779-39-3	Aspafurostanol V:	n.d.	n.d.	n.d.	n.d.	n.d.	n.d.	n.d.	n.d.	n.d.	n.d.	n.d.	n.d.	n.d.	n.d.	n.d.	n.d.	n.d.	n.d.	n.d.	n.d.	n.d.	n.d.
664366-25-0	Aspafurostanol VI	n.d.	n.d.	n.d.	n.d.	2.82±1.82^{a}	2.29±1.04^{a}	2.46±1.77^{a}	n.d.	1.89±1.28	n.d.	n.d.	n.d.	n.d.	n.d.	n.d.	n.d.	n.d.	n.d.	n.d.	n.d.	n.d.	n.d.
Unnasigned-03	Aspafurostanol VII	n.d.	n.d.	n.d.	n.d.	n.d.	n.d.	n.d.	n.d.	n.d.	n.d.	n.d.	n.d.	n.d.	5.50±2.00	12.03±3.75	12.08±3.65	5.42±2.43	7.02±2.44	8.41±3.39	n.d.	n.d.	n.d.
1399745-31-3	Aspafurostanol VIII	n.d.	n.d.	n.d.	n.d.	n.d.	n.d.	n.d.	n.d.	n.d.	n.d.	n.d.	n.d.	n.d.	n.d.	n.d.	n.d.	n.d.	n.d.	n.d.	5.92±1.40^{a}	7.34±2.86^{a}	6.63±1.00^{A}
Unnasigned-07	Aspafurostanol IX	n.d.	n.d.	n.d.	n.d.	n.d.	n.d.	n.d.	n.d.	n.d.	n.d.	n.d.	n.d.	n.d.	n.d.	n.d.	n.d.	n.d.	n.d.	n.d.	3.88±1.66^{a}	6.74±2.80^{a}	5.31±2.02^{A}
Unnasigned-02	Aspafurostanol X	n.d.	n.d.	n.d.	n.d.	n.d.	n.d.	n.d.	n.d.	n.d.	n.d.	n.d.	n.d.	n.d.	n.d.	n.d.	n.d.	n.d.	n.d.	n.d.	6.95±2.87^{b}	12.30±2.92^{a}	9.63±3.78^{A}
Unnasigned-04	Aspafurostanol XI	n.d.	n.d.	n.d.	n.d.	n.d.	n.d.	n.d.	n.d.	n.d.	n.d.	n.d.	n.d.	n.d.	n.d.	n.d.	n.d.	n.d.	n.d.	n.d.	6.90±2.77^{b}	13.96±3.99^{a}	10.43±4.99^{A}
1193356-84-1	Aspafurostanol XI	n.d.	n.d.	n.d.	n.d.	n.d.	n.d.	n.d.	n.d.	n.d.	n.d.	n.d.	n.d.	n.d.	n.d.	n.d.	n.d.	n.d.	n.d.	n.d.	2.85±1.39^{a}	4.20±2.06^{a}	3.52±0.95^{A}
60267-26-7	Asparagoside F	n.d.	n.d.	n.d.	n.d.	5.16±2.88^{ab}	2.01±1.66^{bcd}	5.53±3.07^{ab}	3.21±2.60^{bc}	3.98±1.66^{A}	n.d.	n.d.	n.d.	n.d.	0.96±1.98^{d}	3.01±2.32^{bc}	8.89±2.66^{a}	2.43±1.80^{bc}	2.76±1.88^{bc}	3.61±3.11^{A}	n.d.	1.18±1.80^{cd}	0.59±0.83^{B}
60267-27-8	Asparagoside G	n.d.	1.26±0.05	n.d.	0.42±0.73	n.d.	n.d.	n.d.	n.d.	n.d.	n.d.	n.d.	n.d.	n.d.	n.d.	n.d.	n.d.	n.d.	n.d.	n.d.	n.d.	n.d.	n.d.
84633-34-1	Asparanin B (syn. shatavarin-IV)	9.02±1.77^{a}	13.99±2.23^{a}	10.9±2.09^{a}	11.3±2.51^{A}	1.90±1.95^{b}	n.d.	1.91±1.12^{b}	2.14±1.69^{b}	1.49±1.00^{B}	n.d.	n.d.	n.d.	n.d.	n.d.	n.d.	n.d.	n.d.	n.d.	n.d.	n.d.	n.d.	n.d.
84633-36-3	Asparoside B	1.58±1.23^{a}	2.79±2.62^{a}	1.65±1.56^{a}	2.01±0.68^{A}	0.17±0.34^{b}	1.70±2.01^{a}	0.47±0.35^{b}	0.03±0.59^{b}	0.59±0.76^{B}	n.d.	n.d.	n.d.	n.d.	n.d.	n.d.	n.d.	n.d.	n.d.	n.d.	2.54±2.06^{a}	3.00±1.80^{a}	2.77±0.33^{A}
1493828-40-2	Asparoside B isomer	1.58±1.76^{ab}	2.79±0.78^{a}	1.57±0.72^{ab}	1.98±0.70^{A}	0.17±0.31^{b}	1.70±0.71^{ab}	0.47±0.31^{b}	0.03±0.04^{b}	0.59±0.76^{B}	n.d.	n.d.	n.d.	n.d.	n.d.	n.d.	n.d.	n.d.	n.d.	n.d.	1.12±1.88^{b}	4.20±2.61^{ab}	2.66±2.18^{A}
868560-76-3	Aspaspirostanoside I	0.39±0.25^{c}	1.26±0.34^{b}	2.09±0.11^{a}	1.25±0.85^{A}	n.d.	n.d.	n.d.	n.d.	n.d.	n.d.	n.d.	n.d.	n.d.	n.d.	n.d.	n.d.	n.d.	n.d.	n.d.	n.d.	n.d.	n.d.
Unnasigned-01	Aspaspirostanoside II	6.78±0.98^{b}	3.26±0.45^{c}	2.82±0.76^{c}	4.29±2.17^{A}	n.d.	n.d.	n.d.	n.d.	n.d.	n.d.	n.d.	n.d.	n.d.	n.d.	0.59±1.49^{d}	5.52±1.77^{bc}	1.00±1.43^{d}	10.54±3.00^{a}	3.53±4.49^{B}	n.d.	n.d.	n.d.
84633-33-0	Aspaspirostanoside III	11.09±1.99^{a}	11.35±2.09^{a}	6.68±1.90^{b}	9.71±2.63^{A}	n.d.	n.d.	n.d.	n.d.	n.d.	n.d.	n.d.	n.d.	n.d.	n.d.	n.d.	n.d.	n.d.	n.d.	n.d.	n.d.	n.d.	n.d.
346617-77-4	Aspaspirostanoside IV	0.66±0.23^{h}	n.d.	n.d.	0.22±0.38^{C}	38.8±3.99^{cd}	34.53±5.09^{d}	33.98±4.49^{d}	47.98±5.93^{b}	38.82±6.47	46.41±6.23^{b}	44.49±12.09^{b}	68.18±6.09^{a}	53.03±13.15^{A}	17.63±3.43^{e}	1.80±1.97^{g}	11.22±3.88^{f}	3.56±1.76^{g}	9.85±2.90^{f}	8.81±6.35^{B}	n.d.	n.d.	n.d.
84765-74-2	Aspaspirostanoside V	n.d.	0.16±1.03^{d}	n.d.	0.05±0.19^{B}	6.66±1.46^{a}	6.28±1.80^{a}	5.77±2.09^{ab}	8.52±2.78^{a}	6.81±1.20^{A}	n.d.	2.16±0.76^{cd}	n.d.	0.72±1.25^{B}	5.31±1.76^{b}	5.20±2.04^{bc}	2.34±0.59^{c}	2.93±1.48^{c}	5.85±3.98^{bc}	4.33±1.58^{A}	0.98±1.03^{d}	n.d.	0.49±0.70^{B}
1265882-67-4	Aspaspirostanoside VI:	n.d.	n.d.	n.d.	n.d.	0.78±1.77^{a}	0.67±1.09^{a}	0.41±1.96^{a}	1.07±1.08^{a}	0.73±0.27^{A}	n.d.	n.d.	n.d.	n.d.	n.d.	1.56±0.90^{a}	n.d.	n.d.	n.d.	0.31±0.70^{A}	n.d.	n.d.	n.d.
58881-26-8	Aspaspirostanoside VII:	n.d.	n.d.	n.d.	n.d.	n.d.	n.d.	n.d.	n.d.	n.d.	n.d.	n.d.	n.d.	n.d.	n.d.	n.d.	5.50±2.30	n.d.	n.d.	1.10±2.46	n.d.	n.d.	n.d.
1297292-56-8	Coreajaponin B	n.d.	n.d.	n.d.	n.d.	n.d.	n.d.	n.d.	n.d.	n.d.	3.9±1.08	n.d.	n.d.	1.30±2.25	n.d.	n.d.	n.d.	n.d.	n.d.	n.d.	n.d.	n.d.	n.d.
19057-60-4	Dioscin	n.d.	n.d.	n.d.	n.d.	0.92±1.09^{b}	1.08±1.31^{b}	0.38±1.48^{b}	n.d.	0.59±0.50^{B}	n.d.	n.d.	n.d.	n.d.	n.d.	n.d.	n.d.	0.66±1.99^{b}	n.d.	0.13±0.29^{B}	7.10±2.07^{a}	4.12±2.93^{a}	5.61±2.11^{A}
173356-79-1	Filicinin A	n.d.	n.d.	n.d.	n.d.	n.d.	n.d.	n.d.	n.d.	n.d.	n.d.	n.d.	n.d.	n.d.	18.28±4.56^{a}	4.40±1.55^{c}	9.34±2.00^{b}	6.87±1.05^{bc}	20.12±3.76^{a}	11.80±3.01	n.d.	n.d.	n.d.
Unnasigned-05	Isomer of Asp IV′	n.d.	n.d.	n.d.	n.d.	2.50±0.80^{bc}	6.28±2.09^{a}	2.14±1.87^{bc}	8.52±2.99^{a}	4.86±3.07^{A}	n.d.	1.78±1.00^{bc}	n.d.	0.59±1.03^{B}	5.31±1.89^{a}	5.20±3.09^{ab}	1.99±1.06^{bc}	2.93±1.30^{b}	n.d.	3.09±2.24^{A}	0.98±1.88^{c}	n.d.	0.49±0.70^{B}
Unnasigned-06	Isomer of asparagoside F	n.d.	n.d.	0.48±1.56^{c}	0.16±0.27^{B}	5.76±2.08^{a}	1.96±1.34^{bc}	5.54±2.07^{ab}	3.42±1.95^{ab}	4.17±1.81^{A}	n.d.	n.d.	n.d.	n.d.	2.64±1.78^{bc}	n.d.	n.d.	n.d.	n.d.	0.53±1.78^{B}	2.12±1.63^{bc}	3.24±2.99^{abc}	2.68±0.79^{A}
54522-52-0	Methyl protodioscin	n.d.	n.d.	n.d.	n.d.	n.d.	n.d.	n.d.	n.d.	n.d.	3.15±1.19^{a}	1.25±1.17^{a}	1.98±1.65^{a}	2.13±0.96	n.d.	n.d.	n.d.	n.d.	n.d.	n.d.	n.d.	n.d.	n.d.
57944-19-1	Officinalisnin-II	n.d.	n.d.	n.d.	n.d.	5.76±2.00^{ab}	2.33±1.06^{bc}	5.54±1.96^{ab}	3.34±2.23^{bc}	4.24±1.68^{A}	n.d.	n.d.	n.d.	n.d.	1.34±1.64^{c}	4.23±1.44^{b}	8.99±3.05^{a}	2.27±1.56^{bc}	0.99±1.44^{c}	3.58±3.28^{AB}	0.89±1.20^{c}	3.33±2.98^{bc}	2.11±1.73^{B}
1383539-99-8	Pallidifloside A	n.d.	n.d.	n.d.	n.d.	n.d.	n.d.	n.d.	n.d.	n.d.	n.d.	n.d.	n.d.	n.d.	13.79±2.90^{ab}	18.99±4.56^{a}	9.07±3.00^{b}	13.11±3.66^{ab}	18.28±4.38^{a}	14.65±4.07^{A}	n.d.	n.d.	n.d.
55056-80-9	Protodioscin	1.13±1.77^{c}	1.15±1.43^{c}	1.33±1.65^{c}	1.20±0.11^{B}	n.d.	n.d.	n.d.	n.d.	n.d.	10.16±2.17^{a}	8.89±2.08^{ab}	1.14±0.89^{c}	6.73±4.88^{A}	n.d.	n.d.	n.d.	n.d.	n.d.	n.d.	6.76±3.40^{b}	3.45±2.01^{bc}	5.11±2.34^{A}
60478-69-5	Protoneodioscin	1.76±1.23^{bc}	1.42±1.23^{c}	1.66±1.45^{bc}	1.61±0.17^{B}	n.d.	n.d.	n.d.	n.d.	n.d.	5.22±1.87^{b}	5.19±1.87^{b}	1.96±1.06^{bc}	3.79±2.45^{AB}	n.d.	n.d.	n.d.	n.d.	n.d.	n.d.	9.21±2.88^{a}	2.23±1.94^{bc}	5.72±4.94^{A}
102115-79-7	Pseudoprotodoioscin	1.01±0.34^{b}	0.78±0.21^{b}	1.64±0.61^{b}	1.14±0.44^{B}	n.d.	n.d.	n.d.	n.d.	n.d.	4.89±1.67^{a}	5.45±1.78^{a}	1.15±1.70^{b}	3.83±2.34^{A}	n.d.	n.d.	n.d.	n.d.	n.d.	n.d.	5.90±1.48^{a}	4.56±2.81^{a}	5.23±0.95^{A}
117557-44-5	Pseudoprotoneodioscin	1.25±0.43^{c}	0.98±0.12^{c}	2.01±0.56^{bc}	1.41±0.53^{B}	n.d.	n.d.	n.d.	n.d.	n.d.	2.68±0.87^{bc}	3.69±1.88^{b}	1.08±1.01^{c}	2.48±1.31^{AB}	n.d.	n.d.	n.d.	n.d.	n.d.	n.d.	8.43±2.19^{a}	2.45±1.07^{b}	5.44±4.23^{A}
1374788-87-0	Sarsaparilloside B	1.39±1.25^{b}	2.15±1.11^{ab}	n.d.	1.18±1.09^{A}	n.d.	n.d.	n.d.	n.d.	n.d.	n.d.	0.56±1.79^{b}	n.d.	0.19±0.32^{B}	n.d.	n.d.	n.d.	n.d.	n.d.	n.d.	1.98±1.70^{b}	4.20±3.00^{a}	2.59±2.28^{A}
24332-93-2	Δ20(22)-sarsaparilloside	n.d.	n.d.	n.d.	n.d.	n.d.	n.d.	n.d.	n.d.	n.d.	2.17±0.63	n.d.	n.d.	0.72±1.25	n.d.	n.d.	n.d.	n.d.	n.d.	n.d.	n.d.	n.d.	n.d.

[a]Data represent means ± SD of samples analyzed in triplicate. Differences in saponin amounts were tested according to one-way ANOVA followed by Duncan's test. Within a file, means followed by different lowercase letters are significantly different at $p < 0.05$, and means followed by capital letters represent the ANOVA test effected for mean values of species ($p < 0.05$). n.d., not detected.

Supplementary Table S2. Saponins structures detected in the *Asparagus* shoots analized

Common Name	CAS number	Structure
25-epi-officinalisnin II	1494664-30-0	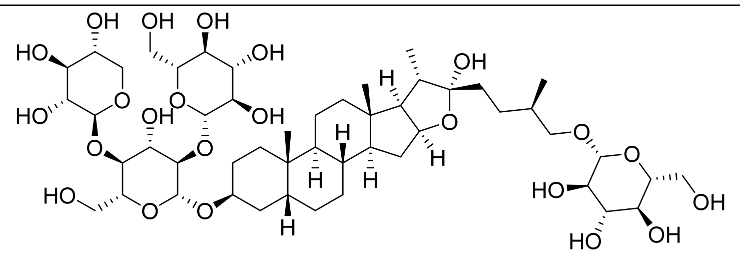<br>ß-D-Glucopyranoside, (3ß,5ß,22a,25R)-26-(ß-D-glucopyranosyloxy)-22-hydroxyfurostan-3-yl O-ß-D-glucopyranosyl-(1→2)-O-[ß-D-xylopyranosyl-(1→4)]<br>1494664-30-0
Aspacochinoside L	2417238-29-8	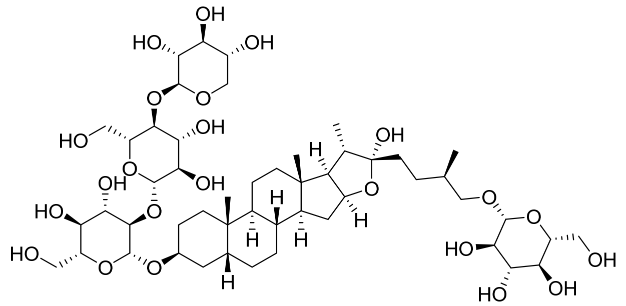<br>Cholestan-22-one, 26-(ß-D-glucopyranosyloxy)-16-hydroxy-3-[(O-ß-D-xylopyranosyl-(1→4)-O-ß-D-glucopyranosyl-(1→2)-ß-D-glucopyranosyl)oxy]-, cyclic 22,16-hemiacetal, (3ß,5ß,16ß,22R,25R)<br>2417238-29-8
Aspacochinoside M	2417238-30-1	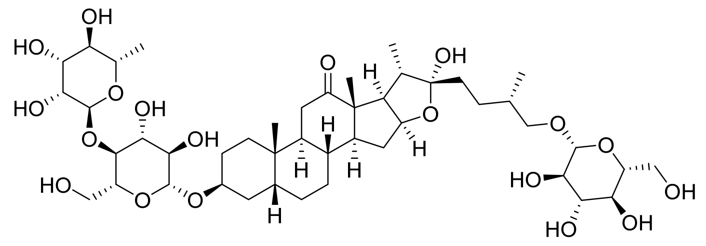<br>Cholestane-12,22-dione, 3-[[4-O-(6-α-L-rhamnopyranosyl)-ß-D-glucopyranosyl]oxy]-26-(ß-D-glucopyranosyloxy)-16-hydroxy-, cyclic 22,16-hemiacetal, (3ß,5ß,16ß,22R,25R)<br>2417238-30-1
Aspacochioside A	557769-32-1	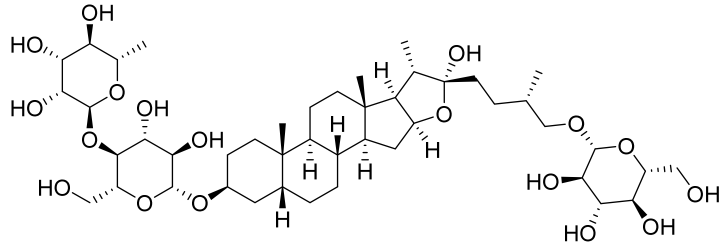<br>ß-D-Glucopyranoside, (3ß,5ß,22a,25S)-26-(ß-D-glucopyranosyloxy)-22-hydroxyfurostan-3-yl 4-O-(6-α-L-rhamnopyranosyl)<br>557769-32-1
Aspacochioside D	1351930-52-3	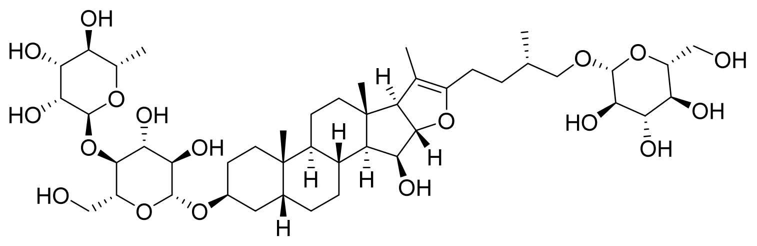<br>ß-D-Glucopyranoside, (3ß,5ß,15ß,25S)-26-(ß-D-glucopyranosyloxy)-15-hydroxyfurost-20(22)-en-3-yl 4-O-(6-α-L-rhamnopyranosyl)<br>1351930-52-3

Aspafilioside C	131123-74-5	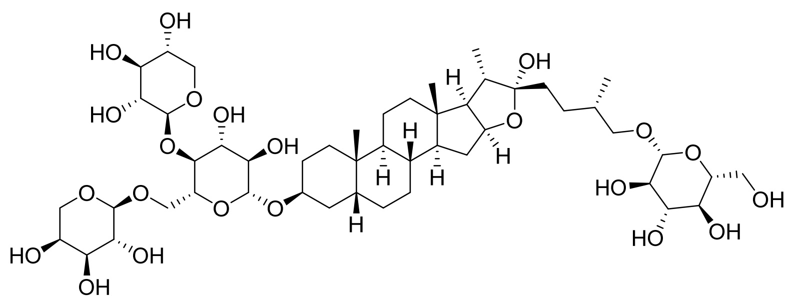<br>ß-D-Glucopyranoside, (3ß,5ß,22a,25S)-26-(ß-D-glucopyranosyloxy)-22-hydroxyfurostan-3-yl O-a-L-arabinopyranosyl-(1→6)-O-[ß-D-xylopyranosyl-(1→4)]<br>131123-74-5
Aspafurostanol I	117457-34-8	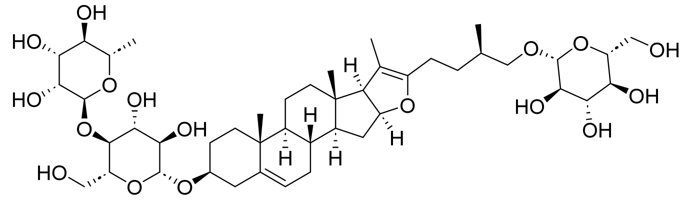<br>3-O-[α-L-Rhamnopyranosyl-(1→4)-β-D-glucopyranosyl]−26-O-β-D-glucopyranosyl-(25-R)-**furostane**-5,20-diene-3β,26-diol<br>117457-34-8
Aspafurostanol II	270926-87-9	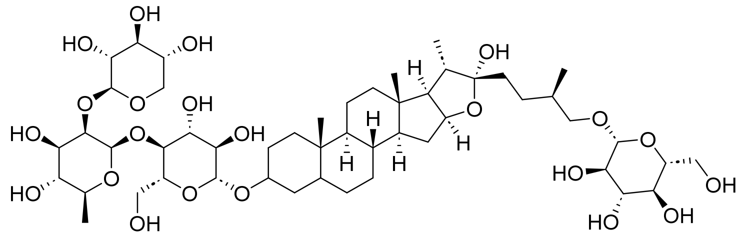<br>(25R)−26-O-β-D-Glucopyranosyl-**furostane**-3β,22α,26-triol-3-O-[β-D-xylopyranosyl-(1→2)]-[α-L-rhamnopyranosyl-(1→4)]-β-D-glucopyranoside<br>270926-87-9
Aspafurostanol III	185432-00-2	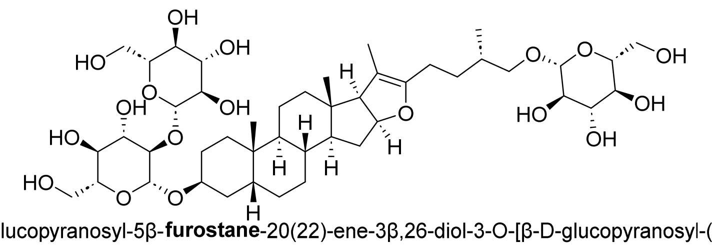<br>Isomer of (25R)−26-O-β-D-glucopyranosyl-5β-**furostane**-20(22)-ene-3β,26-diol-3-O-[β-D-glucopyranosyl-(1→2)]-β-D-glucopyranoside<br>185432-00-2<br>"Anemarsaponin C ó Timosaponin C"
Aspafurostanol IV	1193356-76-1	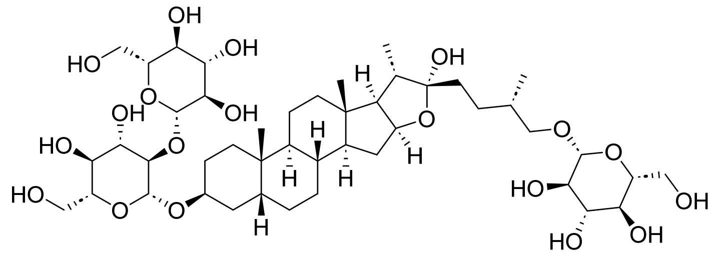<br>3-O-β-D-Glucopyranosyl-(1→2)-β-D-glucopyranosyl-26-O-β-D-glucopyranosyl-(25S)−5β-**furostane**-3β,22α,26-triol<br>1193356-76-1
Aspafurostanol V	244779-39-3	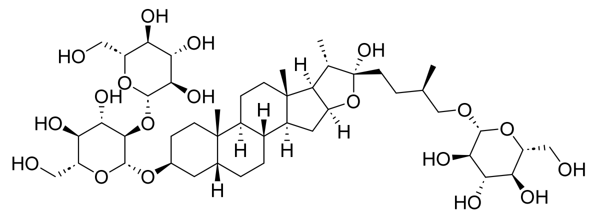<br>3-O-β-D-Glucopyranosyl-(1→2)-β-D-glucopyranosyl-26-O-β-D-glucopyranosyl-(25R)−5β-**furostane**-3β,22α,26-triol<br>244779-39-3

Aspafurostanol VI	664366-25-0	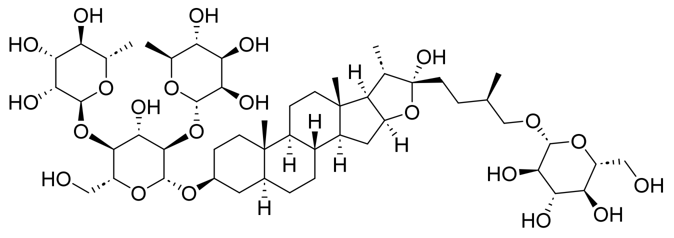<br>3-O-[α-L-Rhamnopyranosyl-(1→4)]-[α-L-rhamnopyranosyl-(1→2)]-β-D-glucopyranosyl-26-O-β-D-glucopyranosyl-5β-**furostane**-3β,22α,26-triol<br>664366-25-0
Aspafurostanol VII	Non-assigned-03	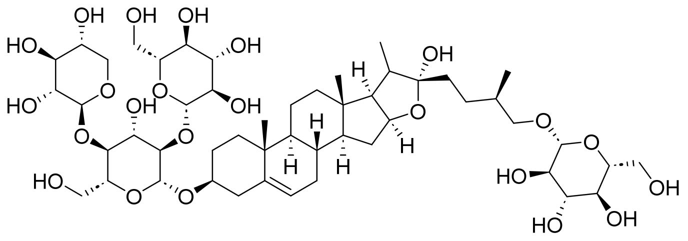<br>26-O-β-D-Glucopyranosyl-**furostane**-5-ene-3β,22α,26-triol-3-O-[β-D-glucopyranosyl-(1→2)]-[β-D-xylopyranosyl-(1→4)]-β-D-glucopyranoside<br>non-assigned-03
Aspafurostanol VIII	1399745-31-3	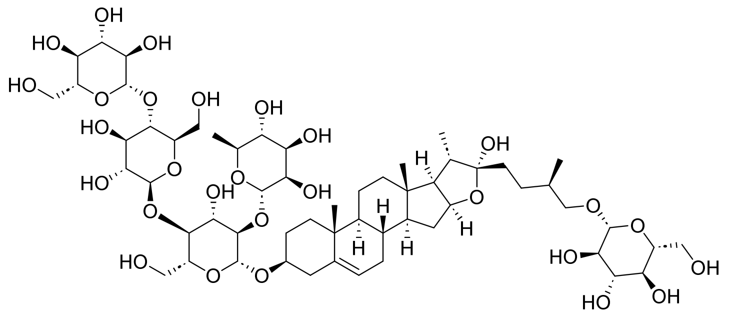<br>26-O-β-D-Glucopyranosyl-**furostane**-5-ene-3β,22α,26-triol-3-O-[α-L-rhamnopyranosyl-(1→2)]-[β-D-glucopyranosyl-(1→4)-α-L-rhamnopyranosyl-(1→4)]-β-D-glucopyranoside<br>1399745-31-3
Aspafurostanol IX	Non-assigned-07	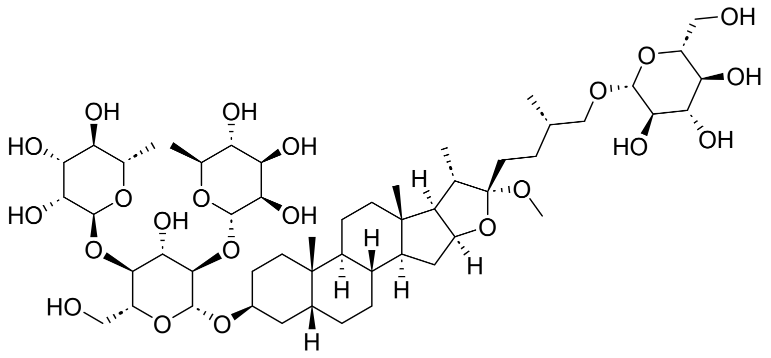<br>Methyl 3-O-[α-L-rhamnopyranosyl-(1→4)]-[α-L-rhamnopyranosyl-(1→2)]-β-D-glucopyranosyl-26-O-β-D-glucopyranosyl-(25S)-5β-**furostane**-3β,22α,26-triol<br>or its isomer<br>non-assigned-07
Aspafurostanol X	Non-assigned-02	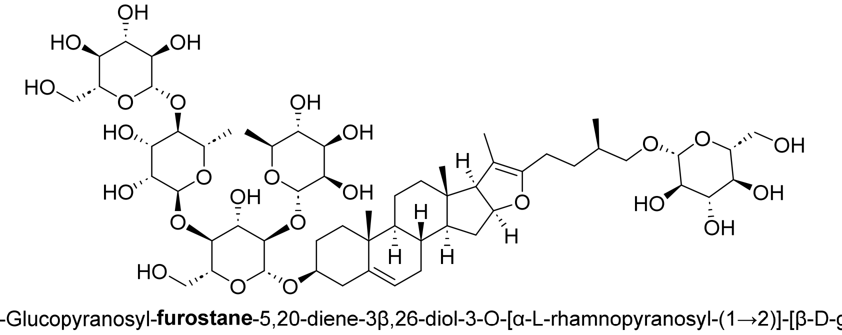<br>(25R)−26-O-β-D-Glucopyranosyl-**furostane**-5,20-diene-3β,26-diol-3-O-[α-L-rhamnopyranosyl-(1→2)]-[β-D-glucopyranosyl-(1→4)-α-L-rhamnopyranosyl-(1→4)]-β-D-glucopyranoside<br>Non-assigned-02

Aspafurostanol XI 1193356-84-1

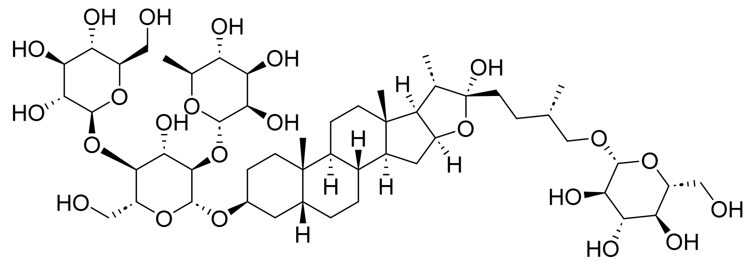

(25S)−26-O-β-D-Glucopyranosyl-5β-**furostane**-3β,22α,26-triol-3-O-[β-D-glucopyranosyl-(1→4)]-[α-L-rhamnopyranosyl-(1→2)]-β-D-glucopyranoside
1193356-84-1

Asparagoside F 60267-26-7

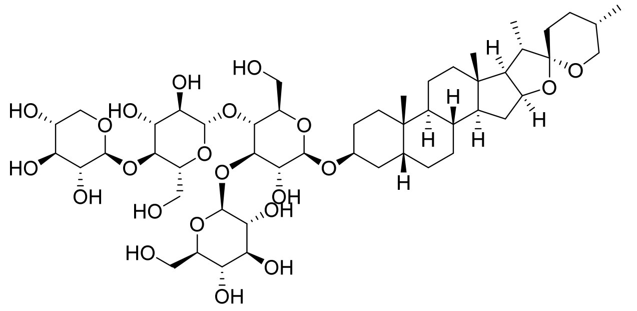

ß-D-Glucopyranoside, (3ß,5ß,25S)-spirostan-3-yl O-ß-D-glucopyranosyl-(1?3)-O-[O-ß-D-xylopyranosyl-(1→4)-ß-D-glucopyranosyl-(1→4)]
60267-26-7

Asparagoside G 60267-27-8

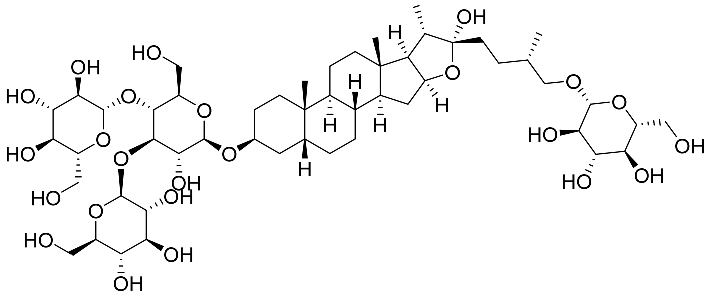

ß-D-Glucopyranoside, (3ß,5ß,22a,25S)-26-(ß-D-glucopyranosyloxy)-22-hydroxyfurostan-3-yl O-ß-D-glucopyranosyl-(1→3)-O-[ß-D-glucopyranosyl-(1→4)]
60267-27-8

Asparoside B 84633-36-3

ó

Asparoside B 301643-62-9

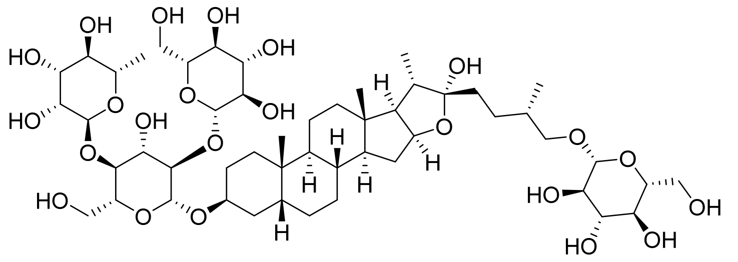

ß-D-Glucopyranoside, (3ß,5ß,22a,25S)-26-(ß-D-glucopyranosyloxy)-22-hydroxyfurostan-3-yl O-6α-L-rhamnopyranosyl-(1→4)-O-[ß-D-glucopyranosyl-(1→2)]
84633-36-3
$C_{51}H_{86}O_{23}$ (Asparagus adscendens)

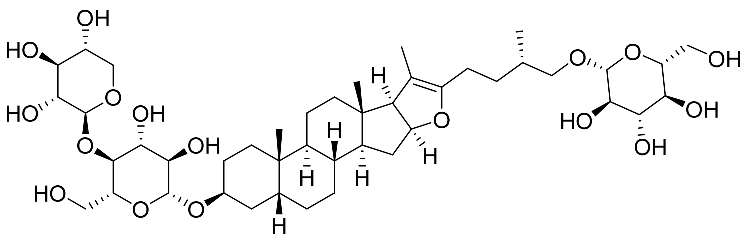

ß-D-Glucopyranoside, (3ß,5ß,25S)-26-(ß-D-glucopyranosyloxy)furost-20(22)-en-3-yl 4-O-ß-D-xylopyranosyl
301643-62-9
$C_{44}H_{72}O_{17}$ (Asparagus meioclados)

Aspaspirostanoside I 868560-76-3

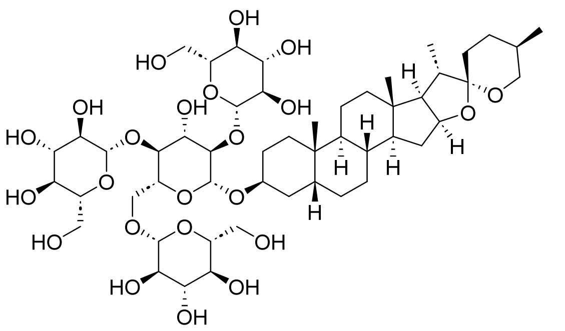

(25R)−5β-**Spirostane**-3β-ol-3-O-β-D-glucopyranosyl-(1→6)-[β-D-glucopyranosyl-(1→2)]-[β-D-glucopyranosyl-(1→4)]-β-D-glucopyranoside
868560-76-3

Aspaspirostanoside II	Non-assigned-01	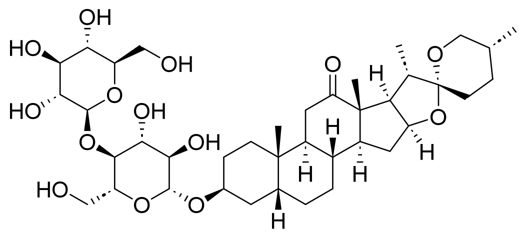<br>(25R)−5β-**Spirostane**-12-keto-3β-ol-3-O-β-D-glucopyranosyl-(1→4)]-β-D-glucopyranoside<br>Non-assigned-01
Aspaspirostanoside III	84633-33-0	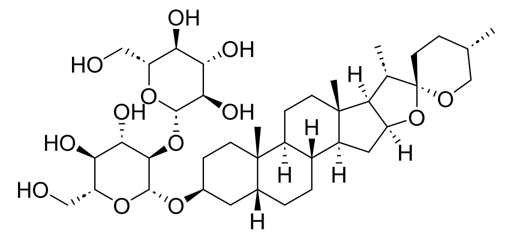<br>(25S)−5β-**Spirostane**-3-O-β-D-glucopyranosyl-(1→2)-β-D-glucopyranoside<br>84633-33-0
Aspaspirostanoside IV	346617-77-4	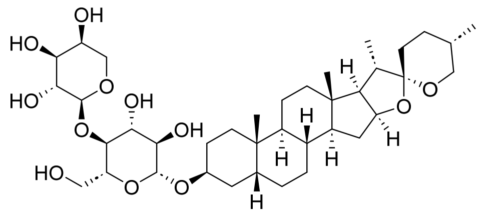<br>(25S)−5β-**Spirostane**-3-O-α-L-arabinopyranosy-(1→4)-β-D-glucopyranoside<br>346617-77-4
Aspaspirostanoside V	84765-74-2	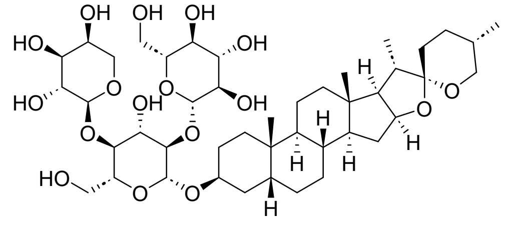<br>(25S)−5β-**Spirostane**-3β-ol-3-O-α-L-arabinopyranosyl-(1→4)-[β-D-glucopyranosyl-(1→2)]-β-D-glucopyranoside<br>84765-74-2
Aspaspirostanoside VI	1265882-67-4	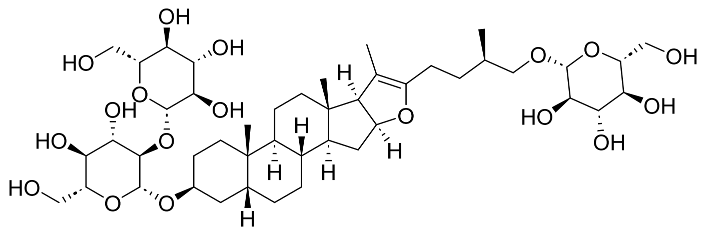<br>(25R)−26-O-β-D-Glucopyranosyl-5β-**furostane**-20(22)-ene-3β,26-diol-3-O-[β-D-glucopyranosyl-(1→2)]-β-D-glucopyranoside<br>1265882-67-4
Aspaspirostanoside VII	58881-26-8	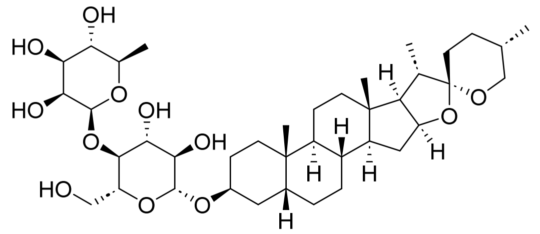<br>(25S)−5β-**Spirostane**-3-O-α-L-rhamnopyranosyl-(1→4)-β-D-glucopyranoside<br>58881-26-8

Coreajaponin B	1297292-56-8	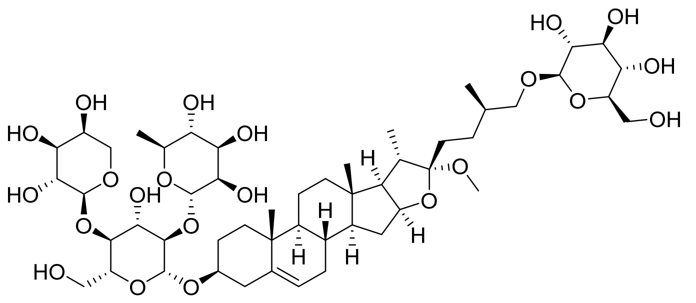<br>ß-D-Glucopyranoside, (3ß,22a,25R)-26-(ß-D-glucopyranosyloxy)-22-methoxyfurost-5-en-3-yl O-a-L-arabinopyranosyl-(1→4)-O-[α-L-rhamnopyranosyl-(1→2)]<br>1297292-56-8
Dioscin	19057-60-4	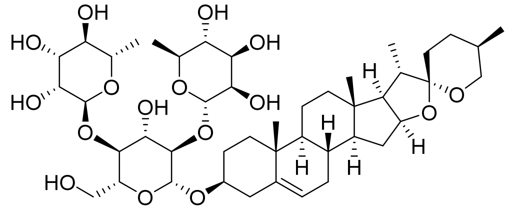<br>ß-D-Glucopyranoside, (3ß,25R)-spirost-5-en-3-yl O-6-α-L-rhamnopyranosyl-(1→2)-O-[6-α-L-rhamnopyranosyl-(1→4)]<br>19057-60-4
Filicinin A	173356-79-1	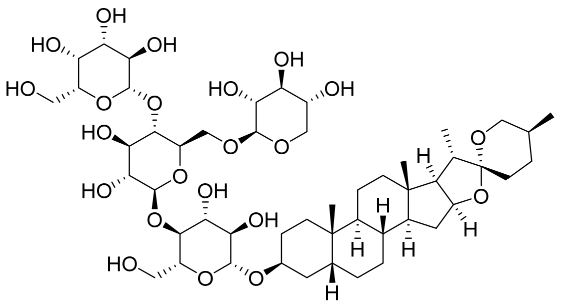<br>ß-D-Glucopyranoside, (3ß,5ß,25S)-spirostan-3-yl O-ß-D-galactopyranosyl-(1→4)-O-[ß-D-xylopyranosyl-(1→6)]-O-ß-D-glucopyranosyl-(1→4)<br>173356-79-1
Isomer of Asp IV′	Non-assigned-05	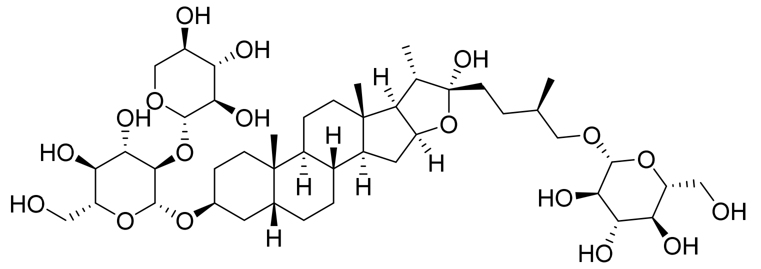<br>ß-D-Glucopyranoside, (3ß,5ß,22a,25R)-3-[[(ß -D-xylopyranosyl)- (1→2)] ß-D-glucopyranosyl]oxy]-22-hydroxyfurostan-26-yl<br>non-assigned-05
Isomer of aspacochioside A	927890-95-7	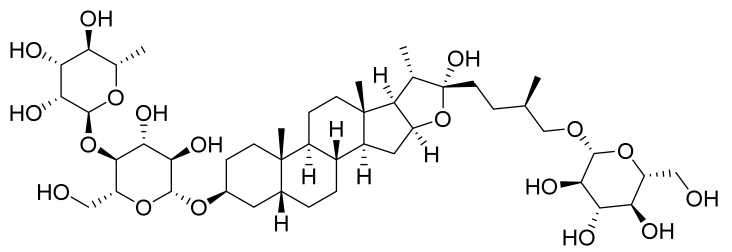<br>ß-D-Glucopyranoside, (3ß,5ß,22a,25R)-26-(ß-D-glucopyranosyloxy)-22-hydroxyfurostan-3-yl 4-O-(6-α-L-rhamnopyranosyl)<br>927890-95-7

Isomer of asparagoside F	Non-assigned-06	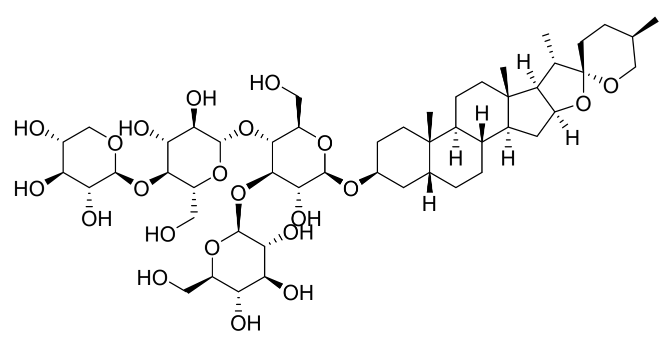<br>ß-D-Glucopyranoside, (3ß,5ß,25S)-spirostan-3-yl O-ß-D-glucopyranosyl-(1→3)-O-[O-ß-D-xylopyranosyl-(1→4)-ß-D-glucopyranosyl-(1?4)]<br>non-assigned-06
Isomer of asparoside B	1493828-40-2	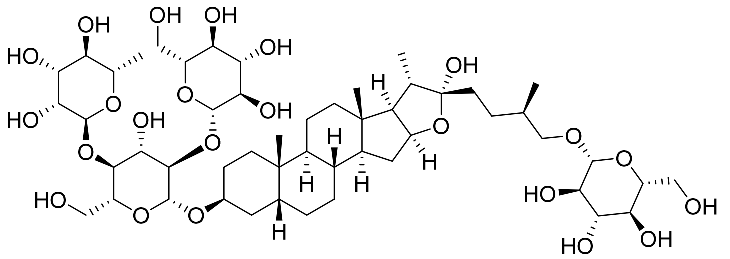<br>ß-D-Glucopyranoside, (3ß,5ß,22a,25R)-26-(ß-D-glucopyranosyloxy)-22-hydroxyfurostan-3-yl O-6-α-L-rhamnopyranosyl-(1→4)-O-[ß-D-glucopyranosyl-(1→2)]<br>1493828-40-2
Methyl protodioscin	54522-52-0	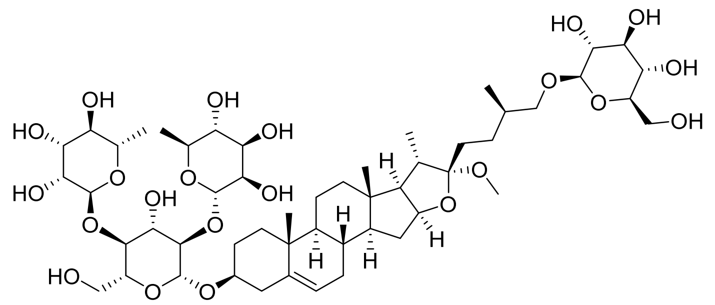<br>ß-D-Glucopyranoside, (3ß,22a,25R)-26-(ß-D-glucopyranosyloxy)-22-methoxyfurost-5-en-3-yl O-6-α-L-rhamnopyranosyl-(1→2)-O-[6-α-L-rhamnopyranosyl-(1→4)]<br>54522-52-0
Officinalisnin-II	57944-19-1	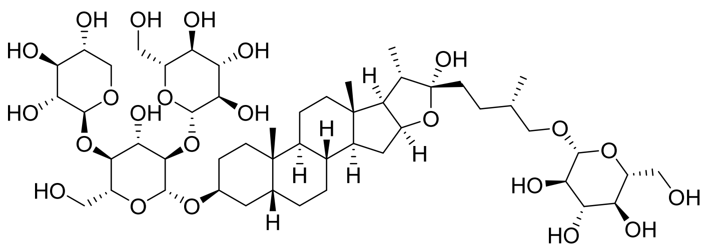<br>ß-D-Glucopyranoside, (3ß,5ß,22a,25S)-26-(ß-D-glucopyranosyloxy)-22-hydroxyfurostan-3-yl O-ß-D-glucopyranosyl-(1→2)-O-[ß-D-xylopyranosyl-(1→4)]<br>57944-19-1
Pallidifloside A	1383539-99-8	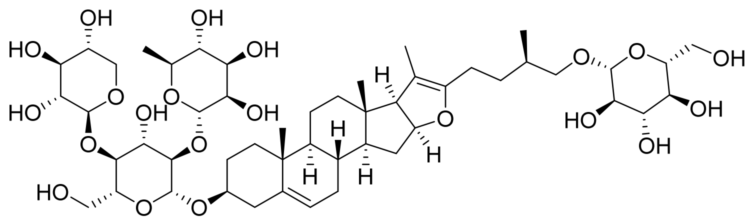<br>ß-D-Glucopyranoside, (3ß,25R)-26-(ß-D-glucopyranosyloxy)furosta-5,20(22)-dien-3-yl O-6-α-L-rhamnopyranosyl-(1→2)-O-[ß-D-xylopyranosyl-(1→4)]<br>1383539-99-8

Protodioscin	55056-80-9	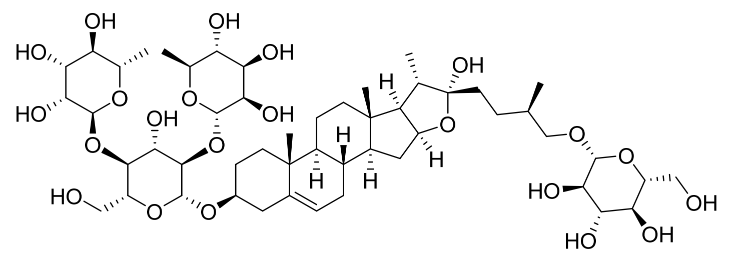<br>ß-D-Glucopyranoside, (3ß,22a,25R)-26-(ß-D-glucopyranosyloxy)-22-hydroxyfurost-5-en-3-yl O-6-deoxy-a-L-mannopyranosyl-(1→2)-O-[6-α-L-rhamnopyranosyl-(1→4)]<br>55056-80-9
Protoneodioscin	60478-69-5	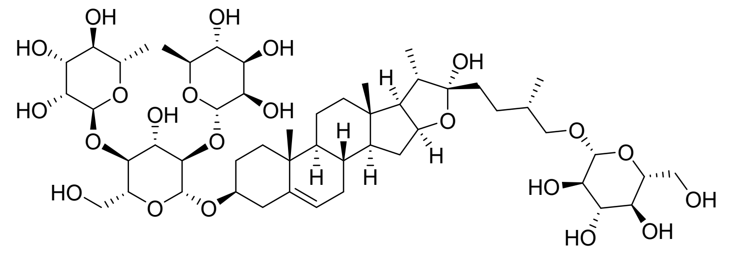<br>ß-D-Glucopyranoside, (3ß,22a,25S)-26-(ß-D-glucopyranosyloxy)-22-hydroxyfurost-5-en-3-yl O-6-α-L-rhamnopyranosyl-(1→2)-O-[6-α-L-rhamnopyranosyl-(1→4)]<br>60478-69-5
Pseudoprotodioscin	102115-79-7	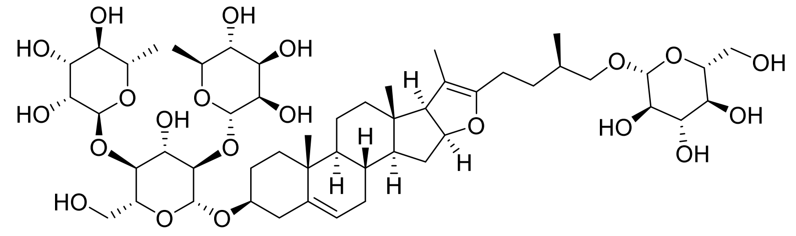<br>ß-D-Glucopyranoside, (3ß,25R)-26-(ß-D-glucopyranosyloxy)furosta-5,20(22)-dien-3-yl O-6-α-L-rhamnopyranosyl-(1→2)-O-[6-α-L-rhamnopyranosyl-(1→4)]<br>102115-79-7
Pseudoprotoneodioscin	117557-44-5	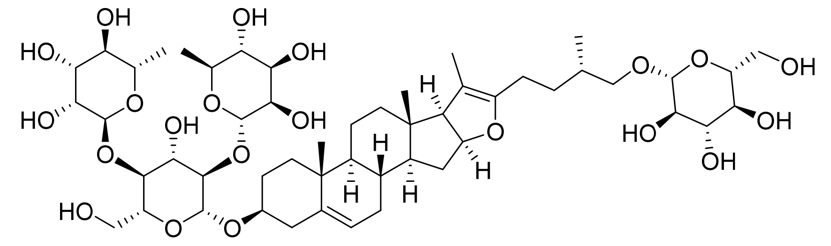<br>ß-D-Glucopyranoside, (3ß,25S)-26-(ß-D-glucopyranosyloxy)furosta-5,20(22)-dien-3-yl O-6α-L-rhamnopyranosyl-(1→2)-O-[6-α-L-rhamnopyranosyl-(1→4)]<br>117557-44-5
Sarsaparilloside	24333-07-1	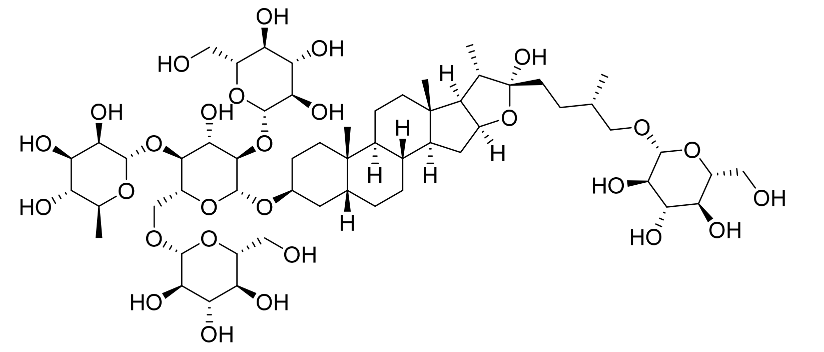<br>ß-D-Glucopyranoside, (3ß,5ß,22a,25S)-26-(ß-D-glucopyranosyloxy)-22-hydroxyfurostan-3-yl O-6-α-L-rhamnopyranosyl-(1→4)-O-[ß-D-glucopyranosyl-(1→2)]-O-[ß-D-glucopyranosyl-(1→6)]<br>24333-07-1

Sarsaparilloside B	1374788-87-0	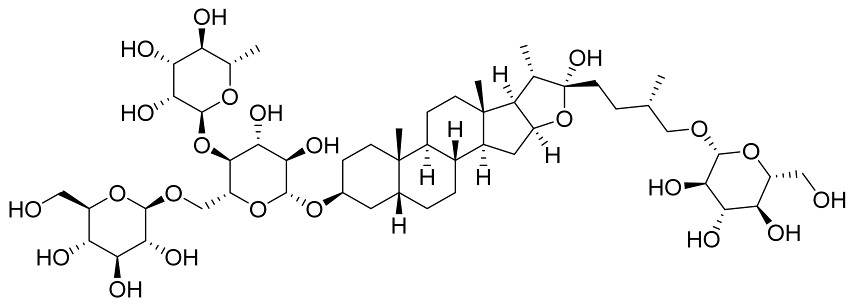<br>ß-D-Glucopyranoside, (3ß,5ß,22a,25S)-26-(ß-D-glucopyranosyloxy)-22-hydroxyfurostan-3-yl O-6-α-L-rhamnopyranosyl-(1→4)-O-[ß-D-glucopyranosyl-(1→6)]<br>1374788-87-0
Shatavarin-IV	84633-34-1	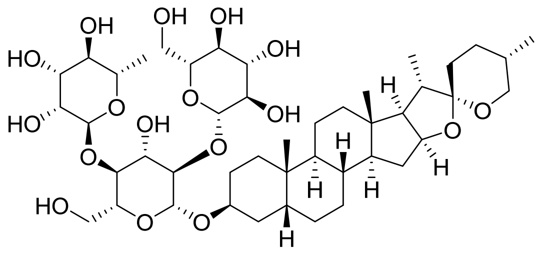<br>ß-D-Glucopyranoside, (3ß,5ß,25S)-spirostan-3-yl O-a-L-arabinopyranosyl-(1→4)-O-[6-α-L-rhamnopyranosyl-(1→6)]-O-[ß-D-glucopyranosyl-(1→2)]<br>84633-34-1
Δ20(22)-sarsaparilloside	24332-93-2	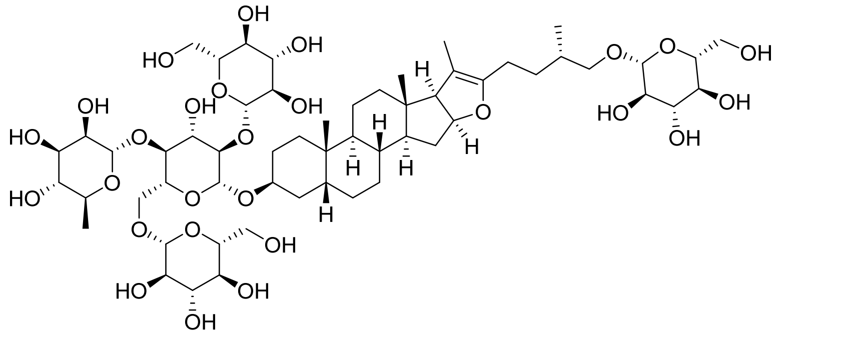<br>Furost-20(22)-ene, 3-[(O-6-α-L-rhamnopyranosyl-(1→4)-O-[ß-D-glucopyranosyl-(1→2)]-O-[ß-D-glucopyranosyl-(1→6)]-ß-D-glucopyranosyl)oxy]-26-(ß-D-glucopyranosyloxy)-, (3ß,5ß,25S)<br>24332-93-2

Supplementary Table S3. Data on saponins determination by LC-MS

CAS Number	Saponin name	Actual RT	Formula	Adduct	*m/z* (Expected)	*m/z* (Delta) ppm
84633-36-3	Asparoside B	10.37	$C_{51}H_{86}O_{23}$	M+H	10.495.527	-159.555
1493828-40-2	Asparoside B isomer	10.37	$C_{51}H_{86}O_{23}$	M+H	10.495.527	-159.555
1374788-87-0	Sarsaparilloside B	10.37	$C_{51}H_{86}O_{23}$	M+H	10.495.527	-159.555
1383539-99-8	Pallidifloside A	10.62	$C_{50}H_{80}O_{21}$	M+H	10.175.264	-201.171
2417238-30-1	Aspacochinoside M	10.65	$C_{45}H_{74}O_{19}$	M+H	9.014.791	-125.759
1297292-56-8	Coreajaponin B	10.66	$C_{51}H_{84}O_{22}$	M-H	1.047.537	-327.498
664366-25-0	Aspafurostanol VI	11.03	$C_{51}H_{86}O_{22}$	M+H	10.335.578	-0.886
270926-87-9	Aspafurostanol II	11.31	$C_{50}H_{84}O_{22}$	M+H	10.195.421	-0.944
131123-74-5	Aspafilioside C	12.3	$C_{45}H_{74}O_{17}$	M+H	8.874.999	0.319
Unnasigned-05	Isomer of Asp IV′	12.86	$C_{44}H_{74}O_{18}$	M+H	8.734.842	-161.642
84765-74-2	Aspaspirostanoside V	12.99	$C_{44}H_{72}O_{17}$	M+H	8.734.842	-245.492
173356-79-1	Filicinin A	16.15	$C_{50}H_{82}O_{22}$	M-H	10.335.214	102.653
346617-77-4	Aspaspirostanoside IV	17.06	$C_{39}H_{62}O_{12}$	M+H	7.114.314	-172.078
868560-76-3	Aspaspirostanoside I	17.08	$C_{51}H_{84}O_{23}$	M+H	10.655.476	-148.233
60267-27-8	Asparagoside G	17.7	$C_{51}H_{86}O_{24}$	M+H	10.655.476	-148.233
1494664-30-0	25-epi-officinalisnin II	17.7	$C_{51}H_{86}O_{24}$	M+H	10.655.476	-148.233
Unnasigned-06	Isomer of asparagoside F	18.4	$C_{50}H_{82}O_{22}$	M+H	10.355.371	-175.916
2417238-29-8	Aspacochinoside L	18.4	$C_{50}H_{84}O_{23}$	M+H	10.355.371	-175.916
60267-26-7	Asparagoside F	18.4	$C_{50}H_{82}O_{22}$	M+H	1.035.537	-166.260
2417238-29-8	Aspacochinoside L	18.4	$C_{50}H_{84}O_{23}$	M+H	10.355.371	-175.916
57944-19-1	Officinalisnin-II	18.4	$C_{50}H_{84}O_{23}$	M+H	1.035.537	-166.260
89590-92-1	Asp VI	18.4	$C_{50}H_{84}O_{23}$	M+H	1.035.537	-166.260
1265882-67-4	Aspaspirostanoside VI:	18.95	$C_{45}H_{74}O_{18}$	M+H	9.034.948	-289.155
185432-00-2	Aspafurostanol III	18.95	$C_{45}H_{74}O_{18}$	M+H	9.034.948	-289.155
Unnasigned-03	Aspafurostanol VII	20.54	$C_{50}H_{82}O_{23}$	M+H	10.335.214	-0.863
Unnasigned-01	Aspaspirostanoside II	24.02	$C_{39}H_{62}O_{14}$	M+H	7.554.212	-161.114
1193356-84-1	Aspafurostanol XI	24.32	$C_{51}H_{86}O_{23}$	M+H	10.495.527	0.382
Unnasigned-07	Aspafurostanol IX	24.87	$C_{52}H_{88}O_{22}$	M+H	10.475.734	171.378
117457-34-8	Aspafurostanol I	25.62	$C_{45}H_{72}O_{17}$	M+H	8.854.842	-180.130
1351930-52-3	Aspacochioside D	25.62	$C_{50}H_{82}O_{21}$	M+H	8.854.825	0.11856
55056-80-9	Protodioscin	25.65	$C_{51}H_{84}O_{22}$	M+H	10.315.421	-176.124
60478-69-5	Protoneodioscin	25.65	$C_{51}H_{84}O_{22}$	M+H	10.315.421	-176.124
102115-79-7	Pseudoprotodoioscin	25.65	$C_{51}H_{82}O_{21}$	M+H	10.315.421	-176.124
117557-44-5	Pseudoprotoneodioscin	25.65	$C_{51}H_{82}O_{21}$	M+H	10.315.421	-176.124
19057-60-4	Dioscin	25.98	$C_{45}H_{72}O_{16}$	M+H	8.694.893	0.583
557769-32-1	Aspacochioside A	25.99	$C_{45}H_{76}O_{18}$	M+H	8.874.998	-197.535
927890-95-7	Aspacochiosidc A isomer	25.99	$C_{45}H_{76}O_{18}$	M+H	8.874.998	-197.535
84633-34-1	Asparanin B (syn. shatavarin-IV)	25.99	$C_{45}H_{74}O_{17}$	M+H	8.874.999	-208.803
58881-26-8	Aspaspirostanoside VII:	26.4	$C_{39}H_{64}O_{12}$	M+H	7.254.471	-212.746
84633-33-0	Aspaspirostanoside III	26.99	$C_{39}H_{64}O_{13}$	M+H	7.414.419	-223.000
24332-93-2	Δ20(22)-sarsaparilloside	29.89	$C_{51}H_{86}O_{22}$	M-H	10.495.527	-206.077
54522-52-0	Methyl protodioscin	30.1	$C_{52}H_{86}O_{22}$	M+H	10.455.578	145.967
1399745-31-3	Aspafurostanol VIII	31.04	$C_{57}H_{94}O_{27}$	M+H	11.935.949	302.510
Unnasigned-02	Aspafurostanol X	31.04	$C_{57}H_{92}O_{26}$	M+H	11.935.949	302.510
Unnasigned-04	Aspafurostanol XI	31.04	$C_{57}H_{92}O_{26}$	M+H	11.935.949	302.510
